# Distinct neuroanatomical and neuropsychological features of Down syndrome compared to related neurodevelopmental disorders: a systematic review

**DOI:** 10.3389/fnins.2023.1225228

**Published:** 2023-08-03

**Authors:** Osama Hamadelseed, Mike K. S. Chan, Michelle B. F. Wong, Thomas Skutella

**Affiliations:** ^1^Department of Neuroanatomy, Institute of Anatomy and Cell Biology, University of Heidelberg, Heidelberg, Germany; ^2^EW European Wellness Academy GmbH, Edenkoben, Germany; ^3^Baden R&D Laboratories GmbH, Edenkoben, Germany; ^4^Stellar Biomolecular Research GmbH, Edenkoben, Germany

**Keywords:** Down syndrome, neuroimaging, neuroanatomy, neuropsychology, fragile X syndrome, Williams syndrome, 22q11.2 deletion syndrome

## Abstract

**Objectives:**

We critically review research findings on the unique changes in brain structure and cognitive function characteristic of Down syndrome (DS) and summarize the similarities and differences with other neurodevelopmental disorders such as Williams syndrome, 22q11.2 deletion syndrome, and fragile X syndrome.

**Methods:**

We conducted a meta-analysis and systematic literature review of 84 studies identified by searching PubMed, Google Scholar, and Web of Science from 1977 to October 2022. This review focuses on the following issues: (1) specific neuroanatomic and histopathological features of DS as revealed by autopsy and modern neuroimaging modalities, (2) language and memory deficits in DS, (3) the relationships between these neuroanatomical and neuropsychological features, and (4) neuroanatomic and neuropsychological differences between DS and related neurodevelopmental syndromes.

**Results:**

Numerous post-mortem and morphometric neuroimaging investigations of individuals with DS have reported complex changes in regional brain volumes, most notably in the hippocampal formation, temporal lobe, frontal lobe, parietal lobe, and cerebellum. Moreover, neuropsychological assessments have revealed deficits in language development, emotional regulation, and memory that reflect these structural changes and are more severe than expected from general cognitive dysfunction. Individuals with DS also show relative preservation of multiple cognitive, linguistic, and social domains compared to normally developed controls and individuals with other neurodevelopmental disorders. However, all these neurodevelopment disorders exhibit substantial heterogeneity among individuals.

**Conclusion:**

People with Down syndrome demonstrate unique neurodevelopmental abnormalities but cannot be regarded as a homogenous group. A comprehensive evaluation of individual intellectual skills is essential for all individuals with neurodevelopment disorders to develop personalized care programs.

## 1. Introduction

### 1.1. The development of Down syndrome

Down syndrome (DS) is the most common chromosomal abnormality associated with intellectual disability (Nadel, 2003). Individuals with DS harbor an extra (third) copy of chromosome 21 in all somatic cells, so DS is also referred to as trisomy 21. The extra copy results from a fertilization event involving an egg or sperm carrying two copies of chromosome 21 due to “non-disjunction” or failure of chromosome separation during meiosis. The cause of non-disjunction is unknown, although studies show it occurs more frequently with maternal age. There are no known environmental or behavioral factors that directly cause non-disjunction.^1^ The DS phenotype is also the same whether the extra chromosome comes from the egg or sperm. Further, there is no evidence that nationality, ethnicity, diet, medication use, illness history, or upbringing influences DS risk. Because trisomy 21 is present from conception, maternal behavior during pregnancy does not affect the disease course. In contrast to maternal age, paternal age appears to have little or no impact on DS risk. Two theories have been proposed to explain the influence of maternal age. According to one theory, all women carry a few eggs with an extra chromosome, which are more likely to be released at the end of reproductive life. Alternatively, the second theory posits that the rate of trisomic conception is the same in all maternal age groups but that impaired pregnancies are more likely to continue in older women (i.e., are less likely to end in miscarriage) due to some mechanism that protects later pregnancies.^2^

### 1.2. Clinical and pathological features of Down syndrome

The clinical characteristics of DS include physical abnormalities, intellectual disabilities, and deficits in emotional regulation. Physical features indicative of DS include a small chin, slanted eyes, poor muscle tone, a flat nasal bridge, a single crease on the palm, a large protruding tongue, and a relatively small mouth. Emotional abnormalities such as depression increase in number and severity with age and may be comorbid with neurological sequela, including epilepsy. For instance, Evenhuis reported generalized tonic–clonic seizures in 6 of 12 moderately intellectually impaired (IQ 35–55) individuals with DS and in all five severely restricted (IQ 25–35) individuals (Evenhuis, 1990). Lai and Williams (1989) also reported markedly elevated incidences of epilepsy (84%) and Parkinsonism (20%) in DS, while Ropper and Williams (1980) reported significantly increased incidences of depression and dementia. Individuals with DS also demonstrate substantial delays and limitations in speech development (Chapman and Hesketh, 2000). Many pathological changes observed in young people with DS are absent *in utero* and emerge during early postnatal development. The proportion of DS individuals with cognitive impairment increases progressively with age, a trend resembling Alzheimer’s disease (AD). These age-related impairments usually begin with slowly progressive memory loss, leading to pervasive cognitive decline accompanied by dementia and emotional changes (Schapiro et al., 1987; Lai and Williams, 1989; Alexander et al., 1997; Nelson et al., 2001). Moreover, senile plaques and neurofibrillary tangles resembling those of the AD brain accumulate in the brains of aging individuals with DS. Further, the regional and temporal patterns of neuropathology are strikingly similar to AD. Therefore, studying the pathogenesis of age-related cognitive dysfunction in DS could help reveal the mechanisms underlying AD and vice versa (Teipel et al., 2003; Hamadelseed et al., 2022). Postmortem studies also show that neurocytological changes in DS are similar to those observed in AD, including multiple neurodegenerative foci. Although AD neuropathology is not an essential aspect of normal aging, there is speculation that DS is associated with accelerated aging (Lott, 1982; Raz et al., 1995).

### 1.3. Neuroanatomical and neuropsychological characteristics of Down syndrome

Down syndrome is the most prevalent hereditary cause of developmental delay, affecting around one out of every 700 live births (Parker et al., 2010). Intellectual disability is the most extensively studied feature of the DS cognitive-behavioral phenotype (Gibson, 1978). However, children with DS have relative strengths and weaknesses that distinguish them from younger, typically developing children and same-age peers with other forms of intellectual disability (Fidler et al., 2008). Children with DS usually display linguistic deficits that surpass general cognitive limits. Furthermore, explicit memory deficits exceed overall intellectual dysfunction (Jarrold et al., 2007; Hamner et al., 2018). However, the precise neuroanatomic correlates of these childhood deficits (and preserved functions) are unclear due to the limited number of post-mortem and neuroimaging studies in infants with DS as well as the small study samples, narrow age ranges, and low spatial resolutions achieved in most previous studies (Pinter et al., 2001a). This research gap is due partly to the challenges associated with properly implementing developmental neuroimaging in this group, such as suppression of movement (Hamner et al., 2018). Nonetheless, neuroimaging research employing sophisticated acquisition and analysis techniques and best practice standards are necessary to achieve progress in this field (Raschle et al., 2012). The goals of this review are to critically evaluate the existing literature on structural neuroimaging of individuals with DS (magnetic resonance imaging [MRI], computed tomography [CT], and (or) voxel-based morphometry [VBM]), particularly studies combining neuroimaging with cognitive–behavioral assessments or comparing cognitive–behavioral phenotypes between DS and other neurodevelopment disorders like fragile X syndrome (FXS), Williams syndrome (WS), and 22q11.2 deletion syndrome (DS22q11.2). It is hoped that synthesis of existing knowledge will provide clues or generate new perspectives on the mechanisms underlying DS-associated brain maldevelopment, the associated intellectual deficits in childhood, adolescence, and adulthood, and age-related AD-like neurodegeneration (Zigman and Lott, 2007; Lee et al., 2015).

## 2. Materials and methods

This study followed the Preferred Reporting Items for Systematic Reviews and Meta-Analyses (PRISMA) guidelines. PubMed, Google Scholar, and Web of Science databases were used to identify relevant peer-reviewed studies of DS published in English and with adequate methodological and statistical information to allow comprehensive critical analysis and replication. We excluded duplicate publications and studies of animal models except for the background. Regarding the excluded 119 articles on “neuroanatomy-brain changes” here, we used the full and restricted exclusion criteria that included only relevant peer-reviewed studies of DS published in English and with adequate methodological and statistical information to allow comprehensive critical analysis and replication and the studies related to this topic represent the greatest number of studies, so the excluded number is expected to be more significant. This review focuses primarily on the following topics: (1) specific brain changes in DS revealed by structural neuroimaging, (2) language and memory deficits as well as preserved functions in DS, (3) the relationship between neuroanatomical and neuropsychological features, and (4) similarities and differences in brain structure and intellectual function between DS and other related neurodevelopmental syndromes such as FXS, WS, and DS22q11.2. These discussions are not restricted by age or gender.

First, we searched for articles with “Brain changes in Down syndrome,” “Neuroanatomy of Down syndrome,” or similar in the title using the keywords “brain changes,” “hippocampus,” “temporal lobe,” “parietal lobe,” “neuroanatomy,” and “Down syndrome.” In addition, we search for articles with “Cognitive profile of Down syndrome,” “Neuropsychology of Down syndrome,” or similar using the keywords “cognitive profile,” “neuropsychology,” “language,” “memory,” and “Down syndrome.” We selected functional and structural neuroimaging studies using CT, MRI, and (or) VBM that also examined the associations of structural changes, especially in the parietal lobe, temporal lobe, and hippocampus, with memory and language function. We also searched for studies with “Comparing brain changes in Down syndrome and Fragile X syndrome,” “Comparing the neuroanatomy of Down syndrome and Fragile X syndrome,” “Comparing brain changes in Down syndrome and Williams syndrome,” “Comparing the neuroanatomy of Down syndrome and Williams syndrome,” “Comparing brain changes in Down syndrome and 22q11.2 deletion syndrome,” “Comparing neuroanatomy of Down syndrome and 22q11.2 deletion syndrome,” or similar in the title using the keywords “comparison,” “brain changes,” “neuroanatomy,” “hippocampus,” “temporal lobe,” “parietal lobe,” “Down syndrome,” “fragile X syndrome,” “Williams syndrome,” and “22q11.2 deletion syndrome.” In addition, we analyzed articles with the titles: “Comparing the cognitive profile of Down syndrome and Fragile X syndrome,” “Comparing neuropsychology of Down syndrome and Fragile X syndrome,” “Comparing the cognitive profile of Down syndrome and Williams syndrome,” “Comparing neuropsychology of Down syndrome and Williams syndrome,” “Comparing the cognitive profile of Down syndrome and 22q11.2 deletion syndrome” “Comparing neuropsychology of Down syndrome and 22q11.2 deletion syndrome” or similar in the title using the keywords “comparison, cognitive profile,” “neuropsychology,” “language,” “memory,” “Down syndrome,” “fragile X syndrome,” “Williams syndrome,” and “22q11.2 deletion syndrome.”

**Figure 1:**
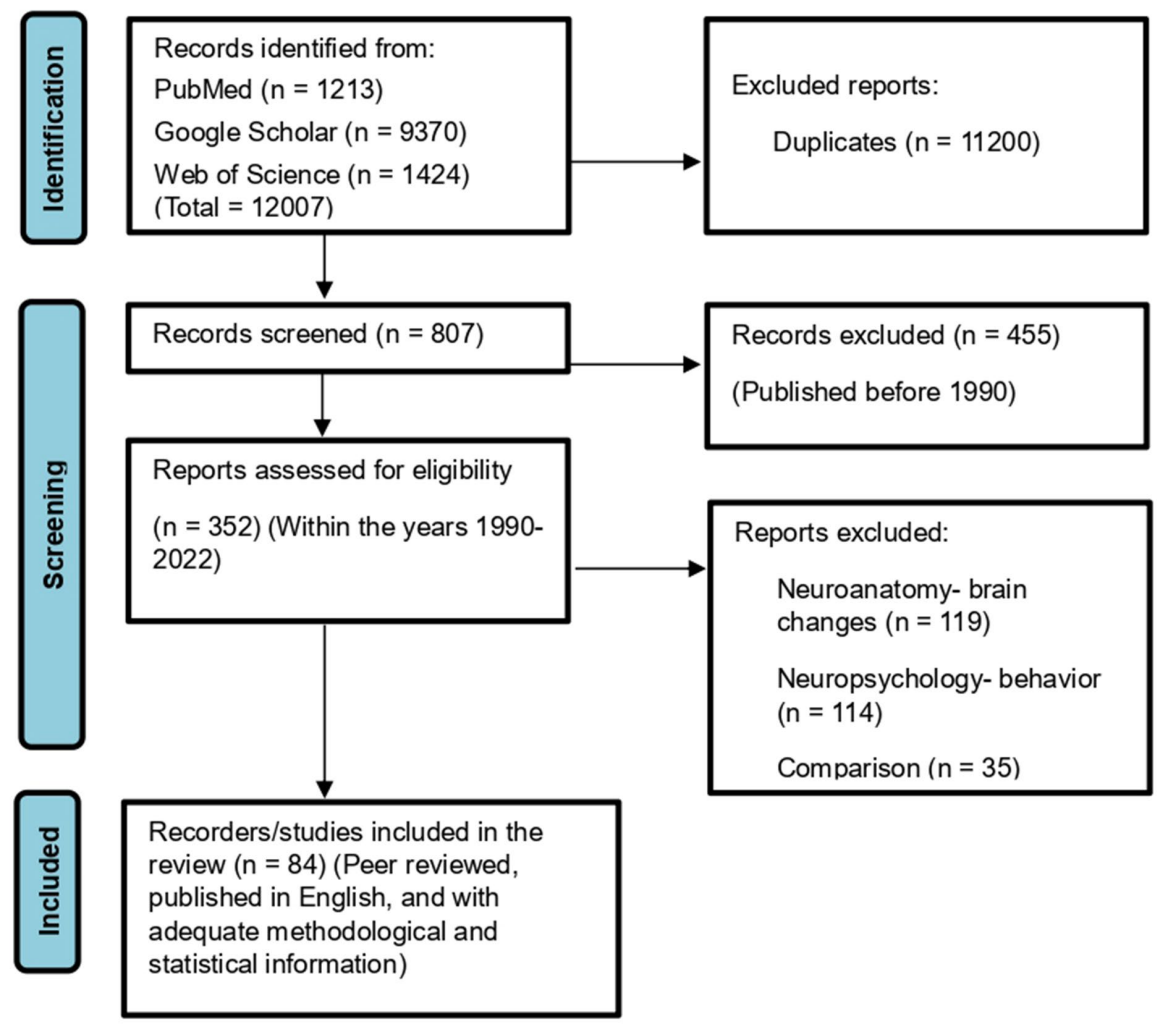
PRISMA 2020 flowchart showing the selection of studies analyzed in this systematic review and meta-analysis.

The meta-analysis was performed on 41 brain structure measurements from 6 studies with sufficient data (e.g., large sample size and broad age range of Down syndrome participants) for the statistical meta-analysis framework. DerSimonian and Laird’s tau estimation was calculated as a standardized mean difference metric for scaling variables, and Cohen’s d was calculated as an estimate of variance among studies (see Table 1). The systematic review is supplemented with descriptive tables, while pooled results of the meta-analysis are presented using forest plots. Funnel plots and Egger’s tests are used to evaluate study publication bias. Eighty four studies were selected in total after using the above-mentioned inclusion and exclusion criteria. A PRISMA flow chart of study inclusion and exclusion is illustrated in Figure 1.

**Table 1:** Descriptive statistics used to express and analyze regional brain volume differences between DS and Control groups in individual studies.

Study	N	DS	Controls	Age group	Structure	Mean (DS)	SD (DS)	Mean (Controls)	SD (Con-trols)
Aylward et al. (1997a)	44	22	22	Adults	Caudate	9.64	1.29	9.96	1.36
					Putamen	10.92	1.71	9.78	1.39
					Globus pallidus	3.27	0.53	3.44	0.73
					Total basal ganglia	23.83	3.09	23.17	2.93
					Brain volume	1078.44	144.44	1260.26	145.2
Frangou et al. (1997)	34	17	17	Adults	Brain volume	945.6	126.1	1155.9	161.8
					Gray matter	363.7	86.6	455.8	82.2
					White matter	382.3	79.5	493.1	115.1
					Lateral ventricles	41.3	30.8	20.7	7.3
Raz et al. (1995)	25	13	12	Adults	Caudate	6.28	0.8	7.39	1.18
					Putamen	9.82	1.03	10.04	1.69
					Globus pallidus	2.02	0.37	2.04	0.45
					Lateral ventricles	14.68	5.16	12.97	7.92
					Precentral gyrus	4.8	0.62	5.3	0.73
					Postcentral gyrus	7.33	0.5	8.68	1.7
					Inferior temporal gyrus	9.11	0.9	11.32	1.85
					The orbitofrontal cortex	5.05	0.94	5.95	1.34
Koenig et al. (2021)	64	37	27	Adults	Cerebral WM	315.131	81.831	415.217	94.079
					Lateral ventricle	9.271	3.399	6.909	2.768
					Total GM	622.305	123.963	735.875	139.052
					Subcortical GM	40.661	4.919	46.818	4.625
					Hippocampus, Left	0.002	0.00018	0.002	0.0001
Weis et al. (1991)	14	7	7	Adults	Caudate	8.6	2.5	8.5	1.7
					Brain volume	1081.6	81	1313.1	146
					White matter	360.3	51.1	462.4	56.1
					Cerebellum	121.3	12.5	149.4	31.2
Pinter et al. (2001a,b)	31	16	15	Pediatric/Young Adults	Brain volume	1068.3	79.7	1297.5	124.2
					Total gray matter	650.2	52.8	773.4	89.3
					Total white matter	418.2	43.1	524.1	51.7
					Total cerebral	951.2	77	1125.3	112.6
					Cerebrum gray matter	572.8	51.7	664.8	78.9
					Subcortical gray matter	43.6	4.6	43.7	4.6
					Cerebrum white matter	378.5	37.8	460.5	48.1
					Frontal lobe	333.2	34.9	402.8	45.5
					Parietal lobe	242.7	21.4	279.8	29.8
					Temporal lobe	187.9	20.3	220.7	24.6
					Occipital lobe	109.8	22.1	131.6	20.9
					Cerebellum	89.4	11	133.8	13.1
					Superior temporal gyrus	31.4	2.8	38.1	4.1

## 3. Results

### 3.1. Neuroanatomical changes in people with Down syndrome

The neuroanatomical changes characteristic of DS can be divided into two categories: (1) those arising from maldevelopment and directly related to the DS genotype and (2) progressive brain atrophy superimposed on neurodevelopmental abnormalities that contribute to AD-like pathology. In recent years, postmortem data on DS neuroanatomy have been supplemented by a growing number of *in vivo* structural neuroimaging studies utilizing CT and MRI (Teipel and Hampel, 2006). We have identified six studies reporting neuroanatomical changes in the brains of people with DS based on neuropathological evidence (see Table 2). Postmortem studies have identified several consistent abnormal features of the DS brain, including reduced total weight, frontal and temporal lobe atrophy, widespread gyral atrophy, cerebral ventricle enlargement, a smaller brain stem, and cerebellar atrophy, particularly in the vermis (Zellweger, 1977; Vogt et al., 1991; Teipel and Hampel, 2006). However, two major factors hamper the interpretation of postmortem studies. First, agonal condition and the long-term pathological changes that lead to death can alter brain anatomy. Second, the lack of information about the patient’s cognitive and developmental status makes selecting a suitable control group difficult. Non-invasive *in vivo* imaging techniques have largely eliminated these limitations. Kesslak et al. (1994) found reduced hippocampal formation volume in a DS group but unexpectedly reported enlargement of the parahippocampal gyrus, possibly reflecting anomalies in neurogenesis throughout development, while differences were found in the volumes of lateral ventricles, combined subcortical nuclear structures, whole neocortex, or temporal gyri between DS and matched control groups. Pinter, Eliez, et al. (2001b) found large volume reductions in frontal and occipital lobes, planum temporale, and superior temporal gyrus. Still, the absolute volume reductions in whole temporal and parietal lobes were no longer significant after adjustment for total brain volume. The relative preservation of temporal lobe volume was associated with greater white matter (WM) volume. According to the authors, this may be due to a specific aberration in WM maturation. However, their technique depended on a semi-automated parcellation of the brain using a normal pediatric template, while individuals with DS may have differently shaped brains, so there may have been methodological challenges in detecting the posterior margins of the temporal lobes on MRI images (Pinter et al., 2001b). Both Jernigan et al. (1993) and Pinter et al. (2001b) found that subcortical gray matter (GM) structures (basal ganglia and thalamus) were normal in DS. Although DS is associated with prenatal defects in neurogenesis and synaptogenesis, the gross brain pathology observed in young individuals is not present *in utero* but occurs during early postnatal development (Zellweger, 1977; Wisniewski, 1990; Vogt et al., 1991). In addition to morphometric changes arising from maldevelopment, evidence from postmortem studies suggests that other morphological changes reflect AD-like neuropathology (Ball et al., 1986; Mann, 1988). Lott postulated that DS is associated with premature aging (Lott, 1982). If so, relatively young people with DS should show neuroanatomical changes characteristic of older normal adults or AD patients. In particular, people with DS, like ordinary older adults and AD patients, would be expected to exhibit some loss of brain tissue, accompanied by pronounced shrinkage of tertiary association areas such as the dorsolateral prefrontal cortex and lower parietal lobule (Haug, 1985; Raz et al., 1993; Kemper, 1994). In addition, persons with DS demonstrated premature shrinkage of the caudate nucleus, mammillary bodies, lobules VI to VIII of the cerebellar vermis, and especially of the hippocampus, all of which have been observed in normal aging, while hippocampal shrinkage is a hallmark of AD (Raz et al., 1992; Kemper, 1994). Raz et al. (1995) investigated these changes in detail by examining neuroanatomical abnormalities of DS and correlations with cognitive changes, while Fernandez-Alcaraz and Carvajal-Molina (2014) reviewed the latest neuroanatomic studies of DS. The reviewed studies indicated that most individuals with DS exhibit a variety of anatomical abnormalities, including brachycephaly and reduced regional brain volumes, particularly in the cerebellum, frontal and temporal lobes, and brainstem. A considerable increase in the volume of the cerebral ventricles was also noted, as well as a smaller hippocampus and amygdala, a narrower superior temporal gyrus, and a decrease in the number and depth of cortical sulci and convolutions (Fernandez-Alcaraz and Carvajal-Molina, 2014). Similarly, distinctive neuropathological signs of AD in the fourth decade of life were reported in the earliest related studies (Wisniewski et al., 1985; de la Monte and Hedley-Whyte, 1990). In addition, the studies reviewed by Fernandez-Alcaraz and Carvajal-Molina (2014) indicated that these anatomical changes occur in similar locations of both hemispheres, including bilateral hippocampus, amygdala, temporal and frontal lobes, and cerebellum, in addition to the brainstem and corpus callosum.

**Table 2:** Summary of studies reporting neuroanatomical changes in the brains of people with DS based on neuropathological evidence.

Study (by the first author)	Year	Findings
Zellweger (1977)		This study described numerous neuroanatomic abnormalities in the DS brain, including reduced total brain volume, ventriculomegaly, and hypoplasia of the cerebellum, temporal lobe, and brainstem
Lott (1982)		This post-mortem neuropathological study reported a modest reduction in brain weight, reduced frontal-occipital diameter, and steeper slopes in both lobes and irregularities of the operculum and superior temporal gyrus.
Mann (1988)		The patterns of senile plaque and neurofibrillary tangle deposition in the brains of individuals with DS over 40 years of age resembled the patterns observed in AD.
Wisniewski (1990)		Autopsy results revealed lower total brain weight, reduced cerebellar volume, a smaller brain stem, a narrower superior temporal gyrus, and fewer neurons per unit area in the brains of the DS group compared to the control group.
Kemper (1994)		The author reported a narrower superior temporal gyrus, a smaller brain stem, and smaller cerebellum in DS.
Fernandez-Alcaraz and Carvajal-Molina (2014)		This review concluded that the hippocampus, amygdala, temporal and frontal lobes, brainstem, cerebellum, and corpus callosum are smaller in the post-mortem DS brain.

DS, Down syndrome; AD, Alzheimer’s disease.

### 3.2. Neuroimaging findings in people with Down syndrome

Until recently, our understanding of the structural brain abnormalities in DS depended on autopsy studies (Fernandez-Alcaraz and Carvajal-Molina, 2014). These studies consistently showed a lower total brain weight, brachycephaly, reduced cerebellar volume, smaller frontal and temporal lobes, simplified sulcal morphology, and a narrower superior temporal gyrus compared to matched controls (Coyle et al., 1986; Wisniewski, 1990; Becker et al., 1991; Vogt et al., 1991). However, post-mortem studies cannot reveal the developmental and pathological mechanisms leading to these differences or control for agonal brain structure changes independent of DS. For these reasons, post-mortem investigations have been largely replaced by *in vivo* neuroimaging (Kates et al., 1997b; Fernandez-Alcaraz and Carvajal-Molina, 2014). There are at least 14 published structural MRI studies on the DS brain, but most have included only subjects older than five years (Jernigan and Bellugi, 1990; Pearlson et al., 1990; Weis et al., 1991; Jernigan et al., 1993; Kesslak et al., 1994; Raz et al., 1995; Aylward et al., 1997a, 1999; Krasuski et al., 2002; White et al., 2003; Beacher et al., 2010; Mullins et al., 2013; Hamner et al., 2018; Koenig et al., 2021). Therefore, the early developmental abnormalities in DS are still largely unknown. Nonetheless, recent improvements in MRI acquisition and processing techniques have enabled higher-resolution quantitative investigations of brain structure in individuals with DS (McCann et al., 2021). We have shown 31 neuroimaging studies reporting anatomical abnormalities in DS in Table 3. In accord with early post-mortem studies, volumetric neuroimaging studies of adults with DS have consistently shown reduced total brain volume and a disproportionately smaller cerebellum, brainstem, frontal lobe, and hippocampal formation (Weis et al., 1991; Kesslak et al., 1994; Raz et al., 1995; Aylward et al., 1997a, 1999; Koenig et al., 2021). In contrast to some post-mortem studies, volumetric MRI studies have reported typical basal ganglion volumes in DS (Raz et al., 1995; Aylward et al., 1997b). Surprisingly, few MRI studies have investigated young children despite the high prevalence of DS among neurodevelopment disorders (Pinter et al., 2001b; Śmigielska-Kuzia et al., 2011; Carducci et al., 2013; Rodrigues et al., 2019). Among the few studies investigating the neuroanatomy of infants and toddlers with DS (Fujii et al., 2017; Gunbey et al., 2017; Levman et al., 2019), a volumetric MRI study by Jernigan et al. (1993) also reported a smaller total brain volume and disproportionately smaller frontal, temporal, and cerebellar regions in six children with DS. Pinter et al. (2001b) assessed brain volumes and tissue composition in DS from early childhood to young adulthood and reported (1) reduced overall brain volume and reduced GM and WM volumes, (2) a disproportionately lower cerebellar volume, and (3) no significant subcortical and cortical volume changes, including in parietal GM and temporal WM, after correction for reduced total GM and WM. These findings are generally consistent with previous pathological and imaging studies (Coyle et al., 1986; Becker et al., 1991; Jernigan et al., 1993; Aylward et al., 1997a). In contrast to studies reporting lower cerebellar and frontal lobe volumes, but consistent with findings from previous studies in adults and children with DS (Jernigan et al., 1993; Raz et al., 1995; Aylward et al., 1997b; Pinter et al., 2001b) found remarkable preservation of subcortical structures, possible due to compensatory subcortical GM adjustment. In addition to increased subcortical GM volumes (Pinter et al., 2001b), others have reported larger lateral ventricles (Pearlson et al., 1998). Automated MRI brain analysis technologies can evaluate cortical thicknesses in different brain regions (Fischl, 2012). To provide a comprehensive evaluation of morphological changes in the DS brain, White et al. (2003) used a fully automated VBM technique on 19 persons with DS living without dementia and 11 age-matched controls. The cerebellum, cingulate gyrus, left medial frontal lobe, right middle/superior temporal gyrus, and left CA2/CA3 area of the hippocampus all showed significant reductions in GM volume (*p* < 0.05, adjusted for multiple comparisons) while WM volume was significantly reduced across the inferior brainstem. In contrast, a superior/caudal section of the brainstem and left parahippocampal gyrus showed statistically significant GM expansion (p < 0.05, adjusted for multiple comparisons), while the bilateral parahippocampal gyrus showed significantly increased WM volume. The DS group also showed substantial cerebral spinal fluid (CSF) volume increases in (larger) lateral ventricles. These findings are consistent with the previous region of interest (ROI)-based imaging investigations of people with DS living without dementia and further contribute to our knowledge of the three-dimensional topography of the DS brain.

**Table 3:** Summary of neuroimaging studies reporting anatomical abnormalities in DS.

Study (by the first Author)	Year	Findings
Ieshima et al. (1984)		This CT study found a relatively small posterior fossa, cerebellum, and brain stem but a relatively larger Sylvian fissure in infants (< 1 year) with DS. CT also revealed a high frequency of midline cava and prominent cisterna magna. In contrast, there were no significant atrophic changes compared to age-matched controls, except after the fifth decade of life.
Schapiro et al. (1987)		According to this quantitative CT study, healthy individuals with DS have smaller brains and lower intracranial volumes than controls. There were no differences in normalized CSF, ventricular, and brain parenchyma volumes between DS cases and controls. Both groups showed significant age-related increases in CSF and ventricular volumes.
Pearlson et al. (1990)		Brain CT scans of individuals with DS revealed a specific temporal pattern of abnormal morphological changes with age compared to controls, including atrophy of the mesial temporal, caudate, anterior subcortical, whole cortical, and whole subcortical regions.
Weis et al. (1991)		Morphometric analysis of MR scans indicated differences in total brain, cerebral cortex, white matter, and cerebellar volumes between DS and control groups, while the volumes of the caudate nucleus, lentiform nucleus, and thalamus did not differ between groups. Brainstem volume was also smaller in DS, but the difference did not reach statistical significance. The whole brain, cerebral cortex, WM, and cerebellar volumes were smaller in the DS group, but there was no regional brain atrophy.
Jernigan et al. (1993)		This study reported smaller frontal cortex, cerebellum, and limbic temporal lobe structures (amygdala, hippocampus, and parahippocampal gyrus) but preserved brainstem volume in DS.
Kesslak et al. (1994)		This MRI study showed reduced hippocampal formation, neocortex, cerebellum, frontal lobe, and temporal lobe volumes, a narrower superior temporal gyrus, and greater ventricular and parahippocampal gyrus volumes. Individuals with DS and clinical dementia exhibited atrophic changes like those typically observed in AD.
Raz et al. (1995)		This study reported smaller cerebral and cerebellar hemispheres, ventral pons, mammillary bodies, and hippocampi in people with DS. Specifically, VI–VIII lobules of the cerebellar vermis were smaller in persons with DS, while there were no differences in other locations. The investigators also found shrinkage of the dorsolateral prefrontal cortex, anterior cingulate gyrus, inferior temporal and parietal cortices, parietal WM, and pericalcarine sulcus in DS participants compared to controls. DS participants exhibited greater parahippocampal gyrus volume but no significant differences in prefrontal WM matter, orbitofrontal cortex, pre- and postcentral gyrus, and basal ganglia volumes. The authors concluded that the morphological brain change patterns in DS were inconsistent with the expectations of premature aging or AD hypotheses.
Aylward et al. (1997a)		Despite reduced total brain volume (TBV), DS participants exhibited larger putamen volumes in this study. There were no differences between DS and control groups between caudate and globus pallidus volumes. These data imply that basal ganglia volume decreases have no role in dementia among older people with DS.
Frangou et al. (1997)		Planum temporale volume was lower in the DS group than in the healthy control group, even after controlling for differences in TBV. In contrast, the relative volume of the superior temporal gyrus did not differ when controlling for TBV.
Aylward et al. (1999)		DS participants without dementia exhibited substantially smaller hippocampi but no difference in amygdalar volume compared to controls after adjustment for TBV. DS participants with dementia exhibited lower hippocampal and amygdalar volumes when controlling for TBV. Neither hippocampal nor amygdalar volume was correlated with age among DS participants without dementia. However, age was correlated with amygdalar volume but not hippocampal volume in people with DS and dementia. Volume changes over time were insignificant in both groups with and without dementia.
Komaki et al. (1999)		This MRI study of DS infants/toddlers aged 2–4 years reported that the pons and cerebellar vermis were much smaller and the cerebral peduncles narrower compared to a matched control group. There was no myelination delay, suggesting pons and cerebellar hypoplasia in DS.
Pinter et al. (2001a,b)		Persons with DS in this MRI study exhibited reduced TBV, with disproportionately smaller cerebellar volumes and proportionally larger subcortical gray matter volumes, in accord with previous imaging investigations. DS participants showed relative preservation of parietal lobe GM and temporal lobe WM volumes compared to control participants. There were no anomalies in brain asymmetry among the DS group.
Krasuski et al. (2002)		This MRI study reported that volumes of the bilateral amygdala, hippocampus, and posterior parahippocampal gyrus decreased with age among individuals with DS.
Teipel et al. (2003)		DS participants showed lower corpus callosum and hippocampal volumes than age-matched healthy individuals, even after controlling for age and total intracranial volume. The DS group exhibited age-related decreases in the corpus callosum area (most noticeable in the posterior region) and hippocampal volume. The effect of age was similar on total corpus callosum and hippocampus volume, while there were no associations between age and corpus callosum or hippocampus volume in the control group.
White et al. (2003)		This VBM study reported GM tissue reductions in the cerebellum, cingulate gyrus, left medial frontal lobe, right middle/superior temporal gyrus, and left CA2/CA3 region of individuals with DS. There were also statistically significant decreases in WM volume across the inferior brainstem and statistically significant GM volume increases in the superior/caudal portion of the brainstem and left parahippocampal gyrus. WM volume was also more significant in the bilateral parahippocampal gyrus of individuals with DS. Further, the authors noted significantly increased CSF volumes suggesting enlarged lateral ventricles in the DS group.
Teipel et al. (2004)		This MRI/VBM study investigated age-related cortical GM alterations in DS individuals without dementia. With advancing age, there was a decline in absolute GM density and volume across the whole brain. GM volume and density were also reduced in the association neocortex, mainly bilateral parietal, left prefrontal, left occipital, and left temporal cortex, in primary sensorimotor areas, and right parahippocampal gyrus when normalized to global GM volume and density. Sex, intracranial volume, and overall cognitive performance did not influence the age effect. Conversely, GM volume and density were preserved in the cerebellum, subcortical nuclei, and basal areas of the frontal and temporal lobes. These findings suggest that localized decreases in GM volume and density in the predementia stage of DS reflect AD-like cortical pathology.
Haier et al. (2008)		This MRI study found reduced GM in the cerebellum, thalamus, caudate, anterior cingulate, frontal lobe, temporal cortex, and hippocampus of individuals with DS at risk of dementia. In contrast, increased GM was found in the pons, superior temporal gyrus, and parahippocampal gyrus.
Beacher et al. (2010)		This MRI study found considerably reduced cranial volume (head size) and TBV in the DS group compared to controls. Specifically, % volume was lower in the left frontal lobe, left and total prefrontal areas, and bilateral cerebellum compared to subjects without DS. Conversely, individuals with DS showed a considerably higher %volume in the bilateral parietal lobe, putamen, lateral ventricles, and left and total occipital lobe. Age-related decreases were found in the whole brain, frontal, temporal, and parietal lobe volumes, while peripheral CSF volume increased with age.
Śmigielska-Kuzia et al. (2011)		This MRI study reported impaired cerebrum development and significant decreases in frontal and temporal lobe, hippocampal, and amygdala volumes compared to controls. There was also a significant TBV reduction in children with DS compared to controls.
Carducci et al. (2013)		This MRI (VBM) study reported reduced TBV in children and adolescents with DS compared to developmentally typical participants. GM volume was also reduced in the cerebellum, frontal lobes, and frontal region of the limbic lobes (cingulate gyri), parahippocampal gyri, and hippocampi of DS children, while GM was preserved in the parietal lobes, left temporal lobe, and right sub-lobar region, including the lentiform nucleus. WM volume was also reduced in the left cerebellum, frontal lobes, parietal lobes, sub-lobar regions, and left brainstem, while WM volume was preserved in the left temporal lobe and temporal areas of the left limbic lobe, including the parahippocampal gyrus. CSF volume surrounding the frontal lobes was also reduced in the DS group.
Lee et al. (2015)		This study found surface area reductions in the frontal lobe, temporal lobe, and superior temporal gyrus, as well as increased cortical thickness in the frontal, parietal, and occipital lobes.
Romano et al. (2016)		This investigation reported strong negative correlations between age and cortical thickness in the frontal, temporal, and parietal lobes and between age and cingulate gyrus volume among participants with DS. Frontal lobe changes were most strongly associated with age. Age-related loss of cortical thickness appeared to be more severe and faster in individuals aged 20 to 30.
Fujii et al. (2017)		This quantitative MRI study reported a smaller brain stem in children with DS compared to the control group. The pons exhibited the most remarkable difference between children with DS and the control group, and the ventral region of the pons showed the most significant mean relative difference.
Gunbey et al. (2017)		Children with DS exhibited significantly lower regional GM volumes in the left putamen, bilateral thalamus superior, caudate nucleus, and cerebellar cortex and reduced WM volumes in the brainstem and corpus callosum. DS participants also exhibited substantially lower subcortical GM volume and total cortical GM volume in both hemispheres and reduced right cerebellar WM volume. There were no significant changes in total WM, total GM, or brain segmentation volumes between DS individuals and controls.
Hamner et al. (2018)		This review presented evidence of reduced TBV, total GM, and WM, cortical lobar, hippocampus, and cerebellar volumes in DS individuals Volume reductions were substantial in the frontal lobe, temporal lobe, cerebellum, and hippocampus, even when corrected for the TBV decrease. One study suggested substantial differences in cortical lobar sizes compared to controls from early childhood to puberty, while group differences were smaller in adolescence. However, frontal lobe GM remains substantially abnormal in the DS group during adolescence. An analysis of age–frontal GM relationships combining effect sizes from published research and data from Lee et al. (2015) revealed a significant negative association between age and frontal GM volume in controls throughout childhood and adolescence but no such association in DS.
Levman et al. (2019)		This MRI study reported increased average cortical thickness in the postcentral gyrus of individuals with DS across all age groups, including Brodmann’s areas 1 and 3b. The authors also found reduced cortical thickness in the lateral orbitofrontal, postcentral gyrus, and other areas of individuals with DS. These findings imply that MRI can identify abnormalities in GM development among individuals with DS.
Rodrigues et al. (2019)		This review concluded that people with DS have reduced TBV, progressive brain atrophy, basal ganglia calcifications, and corpus callosum malformations. Older people with DS also exhibit neuropathological changes characteristic of AD.
Shiohama et al. (2019)		This MRI study reported reduced bilateral cerebellar GM and right cerebellar WM volumes and brainstem and cortical abnormalities (in the right superior temporal, right rostral anterior cingulate, and left rostral middle frontal gyrus) that were independent of comorbidities. Bilateral cerebellar GM and brainstem volumes differed between the DS and healthy groups during infancy.
Patkee et al. (2020)		This MRI assessment of the fetal and neonatal periods found developmental aberrations and abnormal regional brain growth in fetuses with DS from 21 weeks of gestation compared with age-matched controls. The study also reported a reduction in cerebellar volume in the second trimester and a considerable change in cortical growth in the third trimester.
Koenig et al. (2021)		This study reported reduced hippocampal volume in the DS group compared to controls but no association between hippocampal volume and age. Reduced volumes were greatest in CA1 and DG and absent in the subiculum. Reduced intracranial and increased ventricular volumes were also found in individuals with DS, consistent with previous reports. There was a non-significant reduction in cerebral WM volume among individuals with DS but no significant differences in cortical and subcortical GM volumes compared to controls. Cortical thickness was greater, and cortical GM volume was reduced in the DS group. There was an association between age and increased WM.
McCann et al. (2021)		This investigation found greater perirhinal cortex, entorhinal cortex, choroid plexus, and Brodmann’s areas (BA) 3a, 3b, and 44 volumes as a percentage of estimated total intracranial volume (%ETIV) but smaller absolute brain volumes in the DS cohort. The analysis also demonstrated reduced WM volumes in the cuneus, paracentral lobule, postcentral gyrus, and supramarginal gyrus relative to the estimated total intracranial volume.

DS, Down syndrome; CT, computed tomography; MRI, magnetic resonance imaging; CSF, cerebrospinal fluid; AD, Alzheimer’s disease; VBM, voxel-based morphometry; GM, gray matter; WM, white matter; TBV, total brain volume; CA, Cornu Ammon; DG, dentate gyrus; %ETIV, estimated total intracranial volume.

In general, VBM has provided high-resolution quantitative measures of neuroanatomic abnormalities in DS that are generally consistent with prior imaging studies (White et al., 2003). Menghini et al. (2011) identified numerous brain anomalies in a homogenous sample of 12 teenagers with DS compared to healthy controls using VBM, including significantly reduced GM density in the left cerebellum (posterior), right inferior temporal gyrus/medial temporal lobe (fusiform gyrus and hippocampus), and left medial temporal lobe, but greater GM density in the left cerebellum (anterior), right medial temporal lobe (fusiform gyrus), bilateral putamen and caudate nucleus, bilateral insula, bilateral superior frontal gyri, right superior and middle temporal gyri, and bilateral inferior frontal gyri. A similar study by Carducci et al. (2013) of 21 children and adolescents with DS and 27 age-matched controls using MRI-based VBM revealed a smaller whole-brain volume, reduced GM volumes in the cerebellum, frontal lobes, frontal region of the limbic lobe, parahippocampal gyri, and hippocampi, and reduced WM volume in the cerebellum, frontal and parietal lobes, sub-lobar regions, and brainstem of the DS group compared to the control group. In contrast, the GM volumes of the parietal lobes, temporal lobe, and sub-lobar areas, as well as the WM volumes of the temporal lobe and limbic lobe temporal regions, were preserved in DS. Carducci et al. (2013) also reported reduced CSF volume in the frontal lobes. A recent retrospective MRI investigation of individuals with DS aged 0–22 and a cohort of neurotypical participants (aged 0–32) by McCann et al. (2021) reported decreased whole brain, cerebral, cerebellar brainstem, and hippocampal volumes, and increased parahippocampal gyrus volume. Levman et al. (2019) quantitatively assessed the brain morphology of newborns and toddlers with DS using structural MRI and found smaller WM volumes in the right cerebellum, reduced GM volumes in the brainstem and bilateral cerebellum, and morphological abnormalities in the right superior temporal cortex, right rostral anterior cingulate cortex, and left rostral middle frontal cortex. An *in vivo* fetal and neonatal MRI assessment by Patkee et al. (2020) also reported early alterations in cortical and cerebellar growth in DS from 21 weeks of gestation associated with later global declines in whole brain and cortical volumes. Thus, this study detected early biomarkers that may predict the degree of cognitive deficits in DS.

Previous research has focused mainly on children and adults rather than infants with DS, so it is difficult to distinguish between direct pathophysiological effects on the brain and secondary or compensatory processes that occur through living with the disability. However, Patkee et al. (2020) reported that trisomy directly impacts the early *in vivo* growth of the brain and cerebellum, so it may be possible to anticipate the ultimate severity of cognitive deficits for genetic counseling or treatment planning (Patkee et al., 2020). A recent study by Koenig et al. (2021) using a high-resolution 7-T MRI to assess the structure and function of the hippocampus in young adults with DS (mean age 24.5 ± 6.5 years) found reduced whole hippocampal volume, reduced Cornu Ammon (CA) field 1 (CA1) and dentate gyrus volumes, but not significant difference subicular volume. Further, these regional anatomic abnormalities were associated with cognitive deficits (see next section).

In contrast to VMB findings, studies examining regional cortical thickness abnormalities in DS have produced inconsistent results. Lee et al. (2015) and Levman et al. (2019) reported increased cortical thickness in some brain regions of individuals with DS, but Romano et al. (2016) reported decreased cortical thickness. Lee et al. (2015) found that the primary regions affected were the frontal, parietal, and occipital lobes. This study also concluded that people with DS have reduced cortical surface areas in the frontal lobe, temporal lobe, and superior temporal gyrus. Romano et al. (2016) compared the CT scans of older DS participants to those of neurotypical participants as part of a separate study at another imaging center (Levman et al., 2019) and found significant enlargement of CSF spaces, such as in the chiasmatic cistern (Ieshima et al., 1984; Pearlson et al., 1990). Alternatively, Schapiro et al. (1987) found no evidence of age-related ventriculomegaly between DS and normal controls (Schapiro et al., 1987). CT and MRI studies have primarily replicated previously found lower cerebral parenchyma volume (Schapiro et al., 1987). Two MRI studies (Weis et al., 1991; Jernigan et al., 1993) reported that the cerebellum of people with DS was significantly smaller than that of age-matched normal controls, and one (Jernigan et al., 1993) observed global and regional differences in cerebral cortex volume in a small sample of young people with DS compared to normal controls. These two MRI studies (Weis et al., 1991, Jernigan et al., 1993) also showed a preserved average gross volume of the basal ganglia. Fujii et al. (2017) reported a smaller brainstem in children with DS, particularly in the pons, consistent with previous studies (Raz et al., 1995; Komaki et al., 1999). Gunbey et al. (2017) assessed WM integrity and associated WM and GM volumetric changes in early childhood. Haier et al. (2008) reported less GM in brain areas such as the cerebellum, anterior cingulate, frontal lobe, and temporal lobe, including part of the hippocampus. They also reported greater GM in other brain areas such as the pons, superior temporal gyrus, and parahippocampus (Haier et al., 2008).

**Figure 2:**
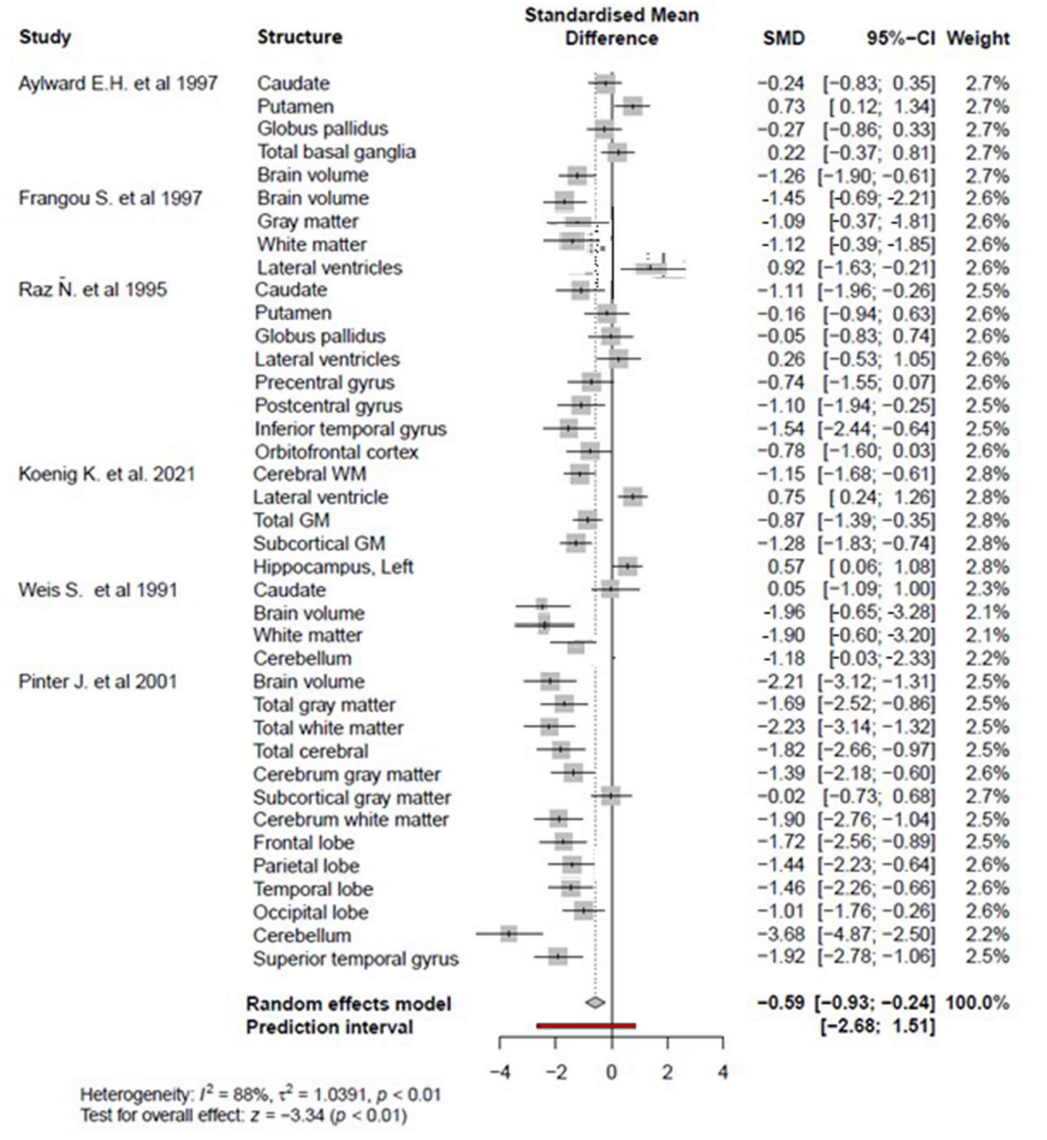
The forest plot for the meta-analysis compares regional brain volumes between individuals with DS and healthy matched controls.

Figure 2 reveals that overall regional brain volume is significantly lower among individuals with DS compared to matched controls (*p* < 0.05) with moderate effect size (overall Cohen’s d = −0.59, 95% CI, −0.93−−0.24) and significant heterogeneity (88%, *p* < 0.01). According to a random effects model, individual brain volumes are shown as gray boxes, and the overall difference is as a diamond. The red bar indicates the 95% Cl of the overall effect. The error bars represent the confidence intervals for individual measures.

**Figure 3:**
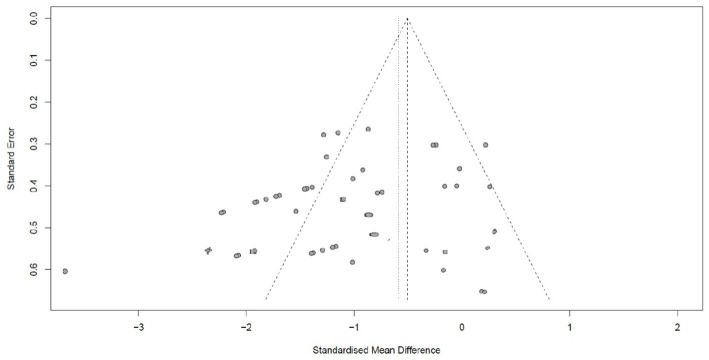
Funnel plot for analysis of publication bias.

There is no or slight publication bias, as evidenced by plot symmetry. Egger’s publication bias test was also non-significant (*t* = −1.23, df = 38, *p* = 0.22) (see Figure 3).

### 3.3. Neuroanatomical changes in the hippocampus of people with Down syndrome

The hippocampus is essential for forming and consolidating explicit memories, including memories for life episodes and semantic information (Squire, 1992), as hippocampal lesions and degeneration lead to catastrophic memory impairments (Isaacs et al., 2003; Savage et al., 2004). The hippocampus changes markedly in size and structure during childhood, with volume peaking between 9 and 11 years of age and decreasing progressively during adolescence (Uematsu et al., 2012). Youth with DS exhibit substantial impairments in hippocampus-dependent memory tasks (Pennington et al., 2003; Clark et al., 2017). Hippocampal atrophy is also a hallmark of AD pathology and a reliable neuroimaging biomarker for the disease (Blanken et al., 2017). Given the AD-like pathology observed in DS, it is surprising that there is little research on hippocampal changes in adolescents with DS (Hamner et al., 2018). Nonetheless, the limited research suggests no significant differences in hippocampal volume between infants with DS and younger typically developing peers (Gunbey et al., 2017). However, children with DS exhibited an absolute reduction in hippocampal volume compared to controls (Śmigielska-Kuzia et al., 2011) and a relative decrease when adjusted for TBV (Pinter et al., 2001a). In addition, VBM studies indicated significant reductions in specific hippocampal subregions in DS during childhood (Menghini et al., 2011; Carducci et al., 2013; Hamner et al., 2018). Carducci et al. (2013) observed a decrease in hippocampal GM volume, especially on the right side, consistent with previous MRI studies of young people with DS (Jernigan et al., 1993; Pinter et al., 2001a) and adults with DS (Raz et al., 1995; Aylward et al., 1997a,b; Pearlson et al., 1998; Pinter et al., 2001b; White et al., 2003). These studies suggest that hippocampal volume reduction in DS begins in childhood and persists throughout adulthood as a direct result of the trisomic genotype (Carducci et al., 2013). Aylward et al. (1999) reported that hippocampus volumes were disproportionately smaller in DS adults with eventual dementia even before dementia onset, while hippocampal volumes did not decrease with age among DS individuals without dementia (Aylward et al., 1999). In contrast, Teipel et al. (2003) found an age-related decrease in hippocampal volumes among DS adults without dementia. Koenig et al. (2021) measured hippocampal subfield volumes and functional connectivity in a group of age-matched normal controls and adolescents and young adults with DS using high-resolution MRI and found significantly reduced total hippocampal volume as well as reduced CA1 and DG volumes, but no change in subicular volume, in the DS group. However, in accord with Aylward et al. (1999), there was no association between age and hippocampal volume. Given the essential function of the subiculum in memory and AD pathology, the lack of volume decrease is unexpected (Koenig et al., 2021). Collectively these observations suggest hippocampal subfield volume reductions in more severely afflicted individuals with DS (i.e., those ultimately developing early onset AD).

### 3.4. Neuroanatomical changes in the temporal and parietal lobes of people with Down syndrome

The parietal and lateral temporal lobes are involved in linguistic and visuomotor skills (Pinter et al., 2001b), both impaired in DS. Kates et al. (2002) reported reduced total parietal and temporal lobe volumes in individuals with DS, while and Pinter et al. (2001b) found an increase in total volumes. Alternatively, a voxel-based whole-brain morphometry study of children and adolescents with DS by Carducci et al. (2013) found symmetrical GM volume maintenance in the right postcentral gyrus, left precuneus, and upper lobule of the parietal lobe, consistent with previous MRI studies on young adults with DS (Jernigan et al., 1993; Pinter et al., 2001b; Kates et al., 2002). However, a few studies of adults with DS have reported reduced parietal lobe volume with aging (Teipel et al., 2004). Conversely, White et al. (2003) found no significant volumetric changes in this region, suggesting that damage to the parietal lobe occurs much later in life. Carducci et al. (2013) also observed a relative decrease in WM volume in the parietal lobes. Moreover, Pinter et al. (2001b) suggested that the deficits in speech and visual processing observed in people with DS are partly due to excessive cell numbers (i.e., insufficient developmental pruning) and concomitant dysfunction of the parietal lobes. The same study by Carducci et al. (2013) also reported preservation of GM volume in the left temporal lobe, while Menghini et al. (2011) found GM loss in the temporal lobe, in accord with previous MRI studies on adults with DS (Kesslak et al., 1994; Raz et al., 1995; White et al., 2003). In addition, postmortem studies by Wisniewski (1990) and Posner and Dehaene (1994) supported late damage to this structure. Thus, early deficits in linguistic and visuomotor skills are not associated with detectable volumetric changes in the parietal and lateral temporal lobes. Rather, these deficits likely reflect disorganization or dysfunction of language circuits within or projecting to these regions.

### 3.5. Hippocampus dysfunction and memory impairment in Down syndrome

Several studies examining hippocampal function in DS have found deficits in both verbal and nonverbal explicit memory (Pennington et al., 2003; Godfrey and Lee, 2018). Impaired verbal short-term memory may significantly impede the development of language skills, particularly vocabulary (Jarrold et al., 2008), and verbal short-term memory is consistently impaired in DS (Pennington et al., 2003). Persons with DS also appear to have impaired explicit long-term memory for verbal information and some deficits in explicit long-term memory for visual object associations (Godfrey and Lee, 2018). In contrast, implicit memory may be less affected in DS, suggesting that neural substrates for this form of memory are relatively well preserved. Moreover, intact implicit memory may provide strategies for improving language development in DS. Impairments in explicit verbal and non-verbal memory support morphometric studies that the hippocampus and functionally associated structures, such as the pre-frontal cortex, are malformed in DS (Jarrold et al., 2008). Pennington et al. (2003) studied hippocampal functions in a sample of 28 school-age children with DS using tasks first developed in animal models and found substantial deficits in hippocampus-dependent memory.

The neurocellular basis of these deficits has been examined in DS mouse models such as Ts65Dn. Hyde and Crnic (2001) reported that hippocampal dysfunction in Ts65Dn mice was influenced by age and associated with reduced numbers of cholinergic hippocampal neurons. In addition, studies of the Ts65Dn mouse model and individuals with DS have suggested that impaired neurogenesis in the molecular layer of the hippocampal dentate gyrus (DG) may contribute to long-term memory deficits (Contestabile et al., 2007). However, the specific contributions of DG neurogenesis to the DS phenotype are still not widely studied (Jarrold et al., 2008).

Given the crucial role of the hippocampus in explicit memory, changes in hippocampal structure and function are a major focus of research investigating biomarkers for AD (Pujol et al., 2018). Neuroimaging studies have shown reduced hippocampal volume associated with memory deficits in the predementia stage of AD among adults with DS (Krasuski et al., 2002; Beacher et al., 2010; Koenig et al., 2021; Hamadelseed et al., 2022). Memory loss is one of the first symptoms of AD (Jahn, 2013) and the temporal order of regional neurodegeneration determines the clinical course and severity of the disease (Hoehne, 2007). Histopathological studies have confirmed that the hippocampus is one of the first and most severely damaged regions by AD (Nagy et al., 1996). Therefore, more detailed structural and functional neuroimaging of the hippocampus may allow for both better prognosis of memory and language impairments in DS and identify the underlying neural substrates, especially in the preclinical stage of DS-associated AD (Godfrey and Lee, 2018; Pujol et al., 2018).

### 3.6. Dysfunction of temporoparietal junction and occipitotemporal structures as markers of deficient language performance in Down syndrome

As discussed earlier, Pinter et al. (2001b) identified morphological changes in multiple temporal lobe structures implicated in speech, although such findings have not been reported consistently across studies. Further, they did not find superior temporal gyrus (STG) volume reduction in DS compared to normal adults with speech deficits, as expected if STG atrophy is involved in DS-associated language dysfunction. They also found no volumetric evidence of an irregular pattern of brain asymmetry (including the temporal lobe) in DS. In contrast, psychological tests examining selective attention and lateralization of brain function within the auditory system suggest lateralized speech dysfunction in DS. While this finding requires additional support from structural and functional neuroimaging studies (Hartley, 1981, 1985; Giencke and Lewandowski, 1989; Pinter et al., 2001b), it appears that language deficits in DS cannot be explained simply by temporal lobe atrophy.

Language function also depends on a supportive network of neural connections that provide related information (Friederici and Gierhan, 2013). Two of these centers are located in the temporal and lower frontal lobes containing Wernicke’s and Broca’s areas (Hamner et al., 2018). Other neural language network structures are located within the temporoparietal junction, occipital lobe (e.g., fusiform gyrus), and superior temporal gyrus (Hickok and Poeppel, 2004; Friederici and Gierhan, 2013; Hamner et al., 2018). The temporoparietal junction structures serve as an auditory–motor interface that transmits heard language to premotor and motor representations in Broca’s area that drive speech output. This hypothesis is consistent with functional imaging evidence that connects perceptual learning to the auditory-motor pathways (Scott and Johnsrude, 2003; Hickok and Poeppel, 2004; Rauschecker and Scott, 2009). High-resolution fMRI and functional connectivity studies are required to identify if any of these pathways are disrupted in DS.

### 3.7. Differences in neuroimaging findings between Down syndrome and fragile X syndrome, Williams syndrome, and chromosome 22q11.2 deletion syndrome (DS22q11.2)

We identified four studies comparing neuroanatomic abnormalities in DS with FXS, WS, and chromosome 22q11.2 deletion syndrome (DS22q11.2) based on imaging data (see Table 4). Previous neuroimaging studies have found an age-related increase in hippocampal volume among fragile X syndrome (SXF) patients compared to a DS group (Reiss et al., 1994; Kates et al., 1997a). In addition, these studies provided evidence of an age-related volume reduction in the superior temporal gyrus, which is essential for speech processing (Reiss et al., 1994). Kates et al. (2002) confirmed these results by studying volumetric changes at the cerebral and lobar levels in young males with FXS (age range: 2–7 years). In addition, they compared persons with the full mutation (FM) and mosaic (Mos) FXS to age-matched controls, individuals with developmental language delay (DLD), and individuals with DS. They found a relative reduction in temporal lobe volume (in the essential GM) but relative preservation or increased parietal lobe WM volume among young individuals with FM FXS (Kates et al., 2002). While the temporal lobe volume reduction was not specific to FXS, as it was also observed in DLD and DS, the parietal lobe preservation/enlargement was specific to FXS.

**Table 4:** Summary of studies comparing neuroanatomic abnormalities in DS with FXS, WS, and chromosome 22q11.2 deletion syndrome (DS22q11.2) based on imaging data.

Author	Year	Findings
Jernigan and Bellugi (1990)		This quantitative MRI study reported a specific pattern of brain dysmorphology in WS distinct from that observed in other forms of intellectual disability, such as DS. In contrast to DS, which frequently is associated with highly regional volume decreases over a large span of the cortex, hippocampus, and cerebellar vermis, individuals with WS exhibited a smaller cerebrum but average cerebellar size. Individuals with WS also displayed a considerably enlarged neocerebellar vermal lobule area and smaller paleocerebellar vermal lobules. WS highly regioselectively impacts brain development, potentially sparing later-developing brain subsystems.
Jernigan et al. (1993)		This MRI investigation of WS patients found that the frontal cortex volume expanded faster than the posterior cortex volume during development. However, overall cerebral volume was reduced in WS compared to controls. In contrast, the frontal cortex is reduced asymmetrically in DS. Compared to other brain structures, limbic regions of the temporal lobe, such as the uncus, amygdala, hippocampus, and parahippocampal gyrus, appear to be spared in WS, while in DS, these structures are smaller than normal. In WS, several subcortical areas, notably the thalamus, putamen, and globus pallidus, show the opposite pattern. Despite a significant decrease in TBV, the volumes of these subcortical structures were average in DS.
Kates et al. (2002)		This study concluded that the magnitudes of volumetric abnormalities are milder in FXS than in DS. Significant absolute and relative reductions in cerebral and lobar volumes among individuals with DS. Cerebral GM volume was substantially reduced, while WM volume reductions were milder. This GM volume reduction was found mainly in the temporal and parietal lobes but not the frontal lobe. Parietal lobe volume reductions appeared to reflect WM shrinkage more than GM shrinkage, while temporal lobe volume reductions involved both GM and WM shrinkage. The superior temporal gyrus was narrower, and the planum temporale volume was smaller in DS. There was also a significant age-dependent reduction in total cerebral tissue, cerebral WM, and temporal GM in DS. Although of a lesser magnitude, similar findings were found in the temporal WM and cerebral GM of individuals with DS.
Koran et al. (2014)		This study reported larger age-related brain changes in people with DS compared to WS and notable changes not observed in WS.

FXS, Fragile X syndrome; WS, William’s syndrome; DS, Down syndrome; MRI, Magnetic resonance imaging; GM, Gray matter; WM, White matter.

Neuroimaging studies of adolescents and adults with Williams syndrome have consistently reported reduced TBV with more significant effects on WM than GM (Jernigan and Bellugi, 1990; Jernigan et al., 1993; Reiss et al., 2000, 2004; Chiang et al., 2007; Koran et al., 2014). In contrast to DS, Meyer-Lindenberg et al. (2005) found largely preserved hippocampal size but subtle changes in shape among adolescents and adults with WS, while a tensor-based morphometry study by Chiang et al. (2007) found reduced parietal lobe volume near the temporoparietal junction and relative preservation of the superior temporal gyrus (Chiang et al., 2007). In contrast, an MRI study by Sampaio et al. (2008) found reduced superior temporal gyrus volume in WS. Further, Campbell et al. (2009) found increased GM volume in the left temporal lobe of children with WS.

Many studies have also examined the brain structure–behavior relations in chromosome 22q11.2 deletion syndrome (DS22q11.2) (Debbané et al., 2006; Kates et al., 2006; DeBoer et al., 2007). Such studies have reported several neuroanatomic abnormalities, including GM and WM changes in the temporal lobe (DeBoer et al., 2007). Eliez et al. (2001) found significant overall brain, superior temporal gyrus, and hippocampal volume reductions in children with DS22q11.2 and schizophrenia and a smaller middle temporal lobe (Eliez et al., 2001). Further, the latter effect was correlated with age. Chromosome 22q11.2 deletion syndrome is considered by some as a genetic subtype of schizophrenia (Zinkstok and van Amelsvoort, 2005). DeBoer et al. (2007) reported a reduction in hippocampal volume in children with DS22q11.2 associated with cognitive impairment, specifically lower Intelligence Quotient (IQ). Zinkstok and van Amelsvoort (2005) also found reduced parietal lobe volume in children and adults with DS22q11.2. Eliez et al. (2000) found loss of normal parietal lobe tissue symmetry due to a significant reduction in left parietal lobe GM among children with DS22q11.2. In addition, Chow et al. (2002) found reduced GM in the left parietal lobe of DS22q11.2 adults associated with schizophrenia symptoms. In contrast, Kates et al. (2002) reported a reduction in parietal WM in DS22q11.2. Finally, Simon et al. (2005) found reduced TBV among children with DS22q11.2 that were associated with a more significant decrease in WM volume than GM volume, consistent with several previous morphometric studies.

In summary, various neuroanatomic abnormalities observed in FXS, WS, and DS22q11.2 are unique to each disorder and distinct from those of DS. However, as with DS, there are few simple direct associations with disease course or symptoms.

### 3.8. Cognitive profile of Down syndrome

We identified 27 studies analyzing the cognitive profile in patients with Down syndrome (see Table 5). Children and adults with DS have numerous intellectual deficits as well as domains of relative preservation or strength, and the profile of strengths and weaknesses can differ markedly among individuals (Wang, 1996; Pulina et al., 2019; Channell et al., 2021; Onnivello et al., 2022). Certain language domains are frequently among the most severely impaired, especially domains related to language expression, while language processing (receptive language function) tends to be less impaired (Abbeduto et al., 2001; Lanfranchi et al., 2009; Pulina et al., 2019). Verbal short-term memory, explicit long-term memory, and executive functions such as working memory, all of which are essential for language development, are also generally impaired in DS (Abbeduto et al., 2001; Chapman R., 2003; Chapman, 2006; Jarrold et al., 2008; Grieco et al., 2015), while visual–spatial short-term memory, implicit long-term memory, perception, and social cognition are preserved or even relative strengths among individuals with DS (Fidler, 2005; Vicari, 2006; Lanfranchi et al., 2009; Martin et al., 2009; Yang et al., 2014; Pulina et al., 2019). These memory deficits are apparent in children with DS compared to developmentally matched controls and include poor immediate verbal memory, simultaneous processing and storage, delayed memory and learning, and gist recall. In contrast, immediate visuospatial memory, semantic information recall, and phonological information retrieval are generally preserved. As a result, children with DS struggle when applying semantic/conceptual information for narrative recall, which is essential for reading development, but do better in phonological information retrieval and semantic/conceptual information retrieval (Conners et al., 2011). The cognitive profile of DS can be assessed using tasks such as the Peabody Picture Vocabulary Test (PPVT), Kaufman Assessment Battery for Children (K-ABC), and Stanford-Binet Intelligence Scale (Chapman, 2006; Pulina et al., 2019).

**Table 5:** Summary of studies analyzing the cognitive profile of Down syndrome.

Author	Year	Findings
Wang (1996)		The study established a cognitive strength and weakness profile for children and young people with DS. Compared to persons with other related developmental disorders, individuals with DS exhibited impaired verbal short-term memory but intact visual-motor abilities. This profile distinguished DS from other cognitive disorders, although dementia may alter this profile in later adulthood.
Chapman and Hesketh (2000)		According to the studies reviewed, the behavioral phenotype of children and adolescents with DS is characterized by developmental delay accompanied by specific deficits in expressive language development, particularly syntax, speech intelligibility, and verbal short-term memory.
Chapman and Hesketh (2000)		This study found expressive language and auditory short-term memory impairments in persons with DS.
Clibbens (2001)		This study presented evidence of delays and chronic difficulties in language morphology and syntax acquisition among children with DS. Language development in children with DS was, on average, delayed relative to overall cognitive, motor, and social development. Both spoken and signed input could help in early language development. These findings demonstrate the importance of establishing joint attention for vocabulary development and indicate that reducing attentional demands may promote language development in children with DS.
Dodd and Thompson (2001)		The study assessed speech in children with DS and compared them to non-DS children with speech impairments (inconsistently producing the same lexical items in the same single-word naming situation). This investigation compared the consistency of real-word creation between groups and found evidence that speech disorders in children with DS are not only caused by intellectual disability but also by physiological aspects of the disease (such as low motor tone and craniofacial deformities). These findings add to the growing body of research showing phonological irregularities in children with DS. The study also found that the number of complete words generated inconsistently by DS children was comparable to the number of inconsistent mistakes made by phonologically disordered children without DS. There were differences in the consistency and quality of inconsistent errors, as intellectually average children with speech disorders made more mistakes involving the addition or deletion of consonants, more changes to the phonemes used on repeated production, and a more comprehensive range of substitutions.
Chapman (2003)		This review concluded that: Infants with DS have learning deficits from 0 to 2 years and are worse by age 4. Language delays (related to cognition) are found in nonverbal request frequency, expressive vocabulary growth, and mean utterance length increase, but not comprehension. Children with DS have selective short-term verbal memory impairments and expressive language deficits relative to comprehension. Adolescents with DS have impaired verbal working memory and delayed recall. The expressive language deficit in syntax is more severe than the expressive language deficit in vocabulary. Up to 50% of people with DS start to show behavioral signs of dementia by age 50. There are age-related increases in depression rates but no link between dementia and higher aggression in people with DS.
Iverson et al. (2003)		Although children with DS exhibited simpler gestural repertoires than linguistic age-matched counterparts, there was no consistent difference in overall gesture use between groups. Individuals with DS created two-element combinations (mainly gesture-word combinations) at a rate equivalent to typically developing counterparts. Children with DS generated no two-word combinations.
Laws and Bishop (2003)		This study evaluated the language profiles of adolescents with DS and children with language impairment matched for the nonverbal cognitive ability to assess differences in correlations between language and other skills. Language profiles were remarkably similar in all areas. Expressive language was more damaged than comprehension, and grammar was more affected than vocabulary in both fields among children with DS. Both groups performed poorly on grammatical morphology and phonological memory tests. The study concluded that there are minor disparities between groups, which may be related to other aspects of DS development.
Pennington et al. (2003)		The study reported an extreme weakness in hippocampal function (e.g., long-term memory) but not prefrontal function (e.g., working memory) relative to overall cognitive function among individuals with DS.
Fidler (2005)		The study reported that some components of visual processing, receptive language, and non-nonverbal social functioning are preserved or relatively strong in DS, while motor abilities and expressive language skills are relatively impaired. Visuospatial processing skills were stronger than verbal processing skills in DS. Strong visuospatial processing was observed in both younger and older children. Visual memory, visual–motor integration, and visual imitation were particularly strong among visuospatial processing domains. In contrast, spatial memory and visuoconstructive task performance were relatively weak in DS. Thus, not all aspects of visuospatial functioning are strong in young children with DS. Many children with DS have substantial linguistic impairments, and many older individuals with DS nonetheless show relative strengths in social functioning
Hick et al. (2005)		This study compared short verbal memory and vocabulary development in children with DS to children with specific language impairments. Both groups demonstrated difficulties in short verbal memory. Throughout the study, children with DS exhibited less progress in verbal short-term memory than children with specific language impairment. Children with DS originally outperformed those with specific language impairments in expressive and receptive vocabulary, but this advantage was not sustained. Children with DS exhibited an advantage in visuospatial short-term memory compared to children with specific language impairments.
Chapman (2006)		The study compared adolescents with DS to adolescents with cognitive impairment of unknown origin. Adolescents with DS exhibited weaker auditory-verbal working memory, comprehension skills, and narrative language competence when there was no opportunity to preview the story. Both groups exhibited strengths in vocabulary comprehension as judged by the Peabody Picture Vocabulary Test-3 relative to nonverbal mental age performance but not in vocabulary comprehension as measured by the Test of Auditory Comprehension of Language-3-vocabulary subtest.
Määttä et al. (2006)		This survey concluded that individuals with DS exhibit delayed cognitive development, including abnormalities in speech, language production, and auditory short-term memory, as well as a higher risk of depression and AD. In contrast, individuals with DS have fewer adaptive behavioral issues than children with other mental disabilities. Children and adolescents with DS show less severe intellectual disability than adults with DS. Younger people with DS exhibit only mild-to-moderate intellectual disability more frequently than those aged 20–39 or over forty.
Vicari (2006)		This review proposed that DS has a complex neuropsychological profile, with some abilities more impaired than others. Motor skills, language (specifically morpho-syntax), verbal short-term memory, and explicit long-term memory are typically impaired, while implicit long-term memory and visual–spatial short-term memory are relatively preserved.
Jarrold et al. (2008)		This study reported that individuals with DS demonstrated poor verbal short-term memory performance and that this deficit would be expected to affect aspects of language acquisition, particularly vocabulary development, negatively. Persons with DS also showed poor explicit long-term memory for verbal information and specific difficulties with explicit long-term memory for visual object associations. Implicit memory, on the other hand, appeared to be less impaired.
Fidler et al. (2008)		The study reported several relative cognitive strengths and weaknesses in children with DS. Visuospatial processing tended to be more robust (consistent with mental age) than verbal processing during childhood and adolescence. Difficulties in verbal processing were associated with working memory and verbal short-term memory deficits. Language delays were expected in DS, but language learning accelerated between the ages of two and four years. Throughout the first few years of life, children with DS developed a profile of relative strengths in receptive versus expressive language, which became more pronounced in early/middle childhood. Syntax was the area of greatest linguistic difficulty for individuals with DS. Persons with DS and autism spectrum disorder exhibited a relative advantage in social development.
Martin et al. (2009)		This review concluded that individuals with DS show a specific language and communication profile, albeit with considerable individual variation. Receptive language is usually superior to expressive language, and vocabulary is more advanced than syntax. Phonology, expressive vocabulary, receptive and expressive syntax, and some pragmatic aspects of language are impaired beyond that expected based on non-nonverbal cognitive deficits. Dementia in older adults with DS may impair various aspects of language and communication. People with DS have relatively strong whole-word recognition abilities.
Lanfranchi et al. (2009)		This study documented deficits in the verbal and central executive (control) components of working memory unrelated to the deficiency in general verbal abilities. Compared to typically developing children with equivalent verbal abilities, central executive function developed more slowly in people with DS. Individuals with DS required additional general resources strictly linked to intelligence to perform well on high-control working memory tasks.
Oliver (2012)		This study observed many deficits in language development among persons with DS. In addition to phonological deficiencies, people with DS demonstrated deficits in expressive vocabulary, syntax (expressive and receptive), and pragmatic abilities. Children with DS exhibited greater spoken language syntax difficulties than expressive and receptive vocabulary problems. Gesture use was a general strength of children with DS. Children with DS had pragmatic, solid skills.
Edgin (2013)		This study reported that people with DS have impaired language and executive functions, including working memory deficits. Previous studies concluded that persons with DS appear to have specific difficulties with morphosyntax. Receptive and expressive vocabulary development and fast mapping were also hampered compared to chronological age-matched controls, but not to the same extent as syntax. Individuals with DS showed less impaired memory for spatial location and more substantial visual attention and perception skills. Implicit memory was less impacted in DS than explicit memory.
Patterson et al. (2013)		This is a systemic review of studies on cognitive development across childhood in DS. The reviewed studies reported poor working memory and reduced long-term memory for explicit information but intact implicit memory. The studies also reported poorer verbal processing and auditory short-term memory skills than expected for mental age but relative strengths in visuospatial processing and non-verbal memory.
Yang et al. (2014)		This review assessed the strength of visuospatial ability relative to general cognitive ability in DS. Individuals with DS demonstrate unequal preservation of the five distinct visuospatial abilities: wayfinding, closure, mental rotation, and visuospatial construction. Persons with DS exhibit no particular “strength in any of the visuospatial skills” relative to general cognitive ability. Visuospatial memory may be weaker than spatial working memory among individuals with DS.
Grieco et al. (2015)		The study reported that nonverbal abilities and social motivation are relative strengths for individuals with DS. Individuals with DS exhibited deficits in language domains, with expressive language, syntax, articulation, phonological processes, and verbal working memory is the most vulnerable.
Godfrey and Lee (2018)		This review concluded that individuals with DS exhibit verbal and nonverbal long-term memory impairments throughout development. There are significant impairments in verbal short-term memory but less consistent effects on nonverbal short-term and working memory.
Pulina et al. (2019)		This review concluded that individuals with DS exhibit greater expressive than receptive language difficulties. Individuals with DS have poor memory spans, particularly auditory-verbal memory spans, and somewhat impaired motor functioning. Nonverbal skills are less affected, and visuospatial processing and social functioning are relative strengths.
Channell et al. (2021)		This study examined phenotypic variability among 314 children, adolescents, and young adults with DS. The authors established three separate categories termed “normative,” “cognitive,” and “behavioral” based on the clustering of strengths and weaknesses. The “normative” class displayed a profile of cognitive and adaptive behavior that was broadly consistent, with relatively low levels of maladaptive behavior, autism spectrum disorder symptomatology, and executive function deficits. The “cognitive” class was distinguished by having the poorest adaptive behavior and lowest performance-based measures of cognition (IQ and visuospatial ability), as well as significant impairments in executive function, maladaptive behaviors, and autism spectrum disorder symptoms. The “behavioral” class showed high rates of maladaptive behavior, notably conduct problems and anxiety, severe executive functioning impairments, and strong autism spectrum disorder symptoms.
Onnivello et al. (2022)		This study reported a more homogenous cognitive profile regarding verbal and nonverbal skills. Participants showed no difference in expressive and receptive vocabulary but did demonstrate differences between verbal and nonverbal domains. There were three distinct profiles: individuals with the lowest scores exhibited the typical DS profile of higher nonverbal than verbal intelligence, those with intermediate scores demonstrated greater verbal than nonverbal intelligence, and those with the highest scores performed equally well in both domains.

DS, Down syndrome; AD, Alzheimer’s disease; IQ, Intelligence Quotient.

### 3.9. Cognitive development in Down syndrome

General intelligence deficits in DS range from moderate to severe (IQ = 25–70) (Pulina et al., 2019). However, a few reports have indicated that some children with DS are in the average IQ range (Chapman and Hesketh, 2001; Vicari, 2006; Vicari et al., 2007; Martin et al., 2009). The IQ scores in DS change with age and are positively or negatively influenced by many genetic and environmental factors influencing typically developing children (Pennington et al., 2003). In addition, AD pathology may be the predominant influence on IQ scores in later life (Bush and Beail, 2004). Longitudinal studies have shown that cognitive development slows during early childhood, and IQ may decrease (Patterson et al., 2013; Sarimski, 2014). Studies monitoring IQ with advancing chronological age have generally found that intellectual disability is mild in children and adolescents compared to adults (Määttä et al., 2006; Vicari, 2006). Couzens et al. (2011) tracked the developmental courses of individuals with DS from childhood to early adulthood using the Stanford Binet Test (4th edition) and found distinct developmental patterns, with crystallized skills (vocabulary, understanding, and quantitative subtests) declining after about 20 years of age. The same trend was also observed for short-term memory. In contrast, fluid abilities (such as pattern analysis) increased rapidly in the first few years of life, followed by constant growth and no decline for at least 30 years (Couzens et al., 2011). A recent literature review by Godfrey and Lee (2018) concluded that long-term memory is impaired from an early age and that verbal short-term memory ability decreases from childhood to adulthood (although there is a lack of IQ studies on young children). Nonverbal short-term and working memory may show a similar trend, although limited data are available.

### 3.10. Language development in Down syndrome

Language and communication skills follow a characteristic pattern in DS. Receptive language is typically better preserved than expressive language, and vocabulary is stronger than syntax, albeit with considerable individual variability (Martin et al., 2009). During development, the language domains most delayed in children with DS are vocabulary, syntax, and morphology, including grammar, verb form, word order, word roots, suffixes, and prefixes (Dodd and Thompson, 2001; Laws and Bishop, 2003; Roberts et al., 2007). Children with DS usually show good pragmatic skills and build a large and varied vocabulary. Typical children with DS also have good social interaction skills and can communicate successfully through language, gestures, and facial expressions (Martin et al., 2009). The use of gestures by children with DS is an important communication skill in the early stages of language development (Iverson et al., 2003). Many studies have reported the effectiveness of gestures and suggested that such use should be promoted to improve social interactions and communication between children with DS and other groups (Launonen, 1994; Miller, 1999; Clibbens, 2001). While there are characteristic patterns of language development, there are certain areas of individual variation. For instance, some children with DS speak their first words at 9 months, whereas others may not speak until several years after birth. Also, word combinations can begin at 18 months in some children with DS but not until school-age in others (Fowler, 1995; Aktas, 2004). According to the National DS Society,^3^ speech problems can also result from difficulties articulating specific sounds, poor mouth or facial muscle tone, sensory processing deficits, phonological processes, and (or) deficits in motor planning for speech. Otitis media, oral anatomy, and atypical facial muscles are all problems that can affect speech development in children with DS (Price et al., 2007; Oliver, 2012). There are also many interventions proposed to improve language skills during the development of children with DS (Hixson, 1993; McWilliam et al., 1996; Chapman R. S., 2003; Dodge, 2004; Fidler, 2005; Hick et al., 2005; McCauley et al., 2006; Paul, 2007; Chapman, 2008). These interventions promote early communication, speaking skills, more complex language and literacy skills, and communication methods such as sign language, images, and symbols (Martin et al., 2009).

### 3.11. Correlations between region-specific hippocampal atrophy and cognitive status in Down syndrome

According to previous neuropathological and neuroimaging studies, hippocampal volumes are disproportionately smaller in DS even before the major declines in cognition observed during development (Wisniewski et al., 1985; Kesslak et al., 1994; Raz et al., 1995; Aylward et al., 1999). Moreover, hippocampal volume decline significantly correlates with the deterioration in memory function, even after controlling for changes in total cognitive score and age (Krasuski et al., 2002; Teipel and Hampel, 2006; Hamadelseed et al., 2022). Two studies also identified atrophy of the hippocampus and parahippocampal gyrus before the onset of dementia in DS (Kesslak et al., 1994; Raz et al., 1995). In agreement with other studies (e.g., Jernigan et al., 1993), both studies showed reductions in hippocampal and parahippocampal gyrus volume in older people with DS and dementia compared to younger individuals with DS but without dementia (Raz et al., 1995; Pearlson et al., 1998; Aylward et al., 1999). Many reports have also used hippocampal atrophy as a marker of cortical neuron loss in AD (Bobinski et al., 1996, 1999; Nagy et al., 1996; Schröder and Pantel, 2016; Hamadelseed et al., 2022; Rao et al., 2022). Several neuroimaging investigations of people with DS but without dementia indicated that the volumes of the hippocampus and neighboring medial temporal lobe structures decrease dramatically with age and that these changes are correlated with early allocortical degenerative alterations and memory loss (Kesslak et al., 1994; Lawlor et al., 2001; Krasuski et al., 2002). However, other studies have found no association between hippocampal volume and age in individuals with DS (Raz et al., 1995; Aylward et al., 1999). A study by Mullins et al. (2013) reported a volume reduction in the hippocampus of people with DS and dementia correlated with cognitive decline. This finding is consistent with studies showing that Mini-mental Status Examination (MMSE) performance correlates directly with hippocampal volume (Ball et al., 1986) and that this MMSE–hippocampal volume association reflects actual atrophy and hippocampal dysfunction (Mullins et al., 2013).

### 3.12. Correlations between region-specific temporal lobe atrophy and language impairment in Down syndrome

Pinter et al. (2001b) found no imaging evidence for disproportionately smaller total temporal lobe volume in DS and found more significant relative (corrected) temporal lobe volume, although the change did not reach statistical significance. Segmenting by tissue type indicated that this relative expansion was due to a significantly larger corrected temporal lobe WM volume. However, it remains unclear if these temporal lobe WM volume changes are associated with cognitive decline in DS, particularly language impairments. Such a relationship may be expected based on functional MRI studies showing significant superior temporal gyrus (STG) activation in healthy adults during auditory and language processing and the prominent language deficits in children with DS (Démonet et al., 1992; Price et al., 1992; Schlosser et al., 1998; Pinter et al., 2001b). Although, Pinter et al. (2001b) did find that corrected STG WM volume was significantly smaller in the DS group, there was no significant difference after adjustment. Krasuski et al. (2002) also reported reduced medial temporal lobe volume and correlations with overall cognitive and memory functions but no correlation with language ability. Menghini et al. (2011) reported reduced regional GM density in the lateral and medial temporal lobes of individuals with DS compared to controls and refined the location of this GM density reduction to the bilateral fusiform gyrus. These results suggest that the right middle temporal gyrus contributes to morphosyntactic ability, and indeed high GM density in this region is positively associated with better morphosyntactic production (Menghini et al., 2011). Finally, Mullins et al. (2013) reported reduced temporal lobe volume in people with DS and dementia and further found a correlation between this volume reduction and cognitive status, including language skills.

### 3.13. Distinct cognitive-behavioral phenotypes of Down syndrome, fragile X syndrome, Williams syndrome, and microdeletion syndrome 22q11

We identified 20 studies that compared cognitive-behavioral phenotypes of DS with FXS, WS, and chromosome 22q11.2 deletion syndrome (DS22q11.2) (see Table 6). Previous studies on FXS have described delayed language development, relative weaknesses in executive function, visual–spatial working memory, perception, and thinking, visual-motor coordination, and short-term memory, but relative strengths in verbal thinking skills and simultaneous processing tasks (Powell et al., 1997; Roberts et al., 2007; Kogan et al., 2009). Further, some of these studies reported sex differences in FXS-related intellectual impairments (Cornish et al., 2007; Huddleston et al., 2014). Males with FXS demonstrated substantial abnormalities in most verbal and visuospatial domains of working memory (Cornish et al., 2007), as well as in memorizing and recounting stories, abilities crucial for developing reading skills (Conners et al., 2011), while delayed memory was relatively less impaired. Rapid phonological recall and semantic/conceptual memory are also crucial aspects of reading development, particularly for young males, but relatively little is known about such capacities in FXS (Conners et al., 2011). The cognitive profile of DS appears distinct from that of FXS in several respects, including more severe language deficits in DS but better reading skills, visual memory, visual perception, and visuomotor skills than among individuals with FXS.

**Table 6:** Summary of studies comparing cognitive-behavioral phenotypes of DS with FXS, WS, and chromosome 22q11.2 deletion syndrome (DS22q11.2).

Author	Year	Findings
Jernigan et al. (1993)		This study reported poor visuospatial and visuomotor abilities but relatively intact language skills in WS. It observed equally severe language deficits in DS and WS. Lexical knowledge, word fluency, and syntax measures were relative strengths in WS. Deficits in visuospatial domains were similar to DS.
Powell et al. (1997)		Participants with FXS performed poorly on tests of sequential processing compared to simultaneous processing, a difference not observed in the DS group. There were significant differences in individual subtest scores within the domain of simultaneous processing for the FXS group but not the DS group. The DS group scored higher on reading skills than the FXS group.
Abbeduto et al. (2001)		This study reported impaired receptive language, expressive language, and social functions (Theory of Mind) in DS, with receptive language deficits more severe than nonverbal cognitive deficits. Also, receptive and expressive language deficits were more severe in DS than in FXS.
Vicari (2001)		This evaluated implicit memory processes in children with WS compared to children with DS and typically developing (TD) children of the same age. Unique memory patterns were found in DS compared to WS. Explicit memory ability in WS was similar to typically developing mental-age counterparts, while participants with DS exhibited poorer explicit memory than either WS or TD children. Implicit learning and memory domains also differed between WS and DS groups. Performance on repeated priming tasks did not differ between WS and DS, but persons with WS were hindered in learning new procedures.
Ypsilanti et al. (2005)		The study reported significant differences in linguist task performance between DS and WS, with the WS group performing better on an unusual word definition task.
Rice et al. (2005)		The study reported that children with FXS have delayed speech and language abilities, with language delays paralleling nonverbal cognitive delays. Children with FXS demonstrated relative strengths in verbal abilities compared to visual–spatial cognitive tasks, similar to children with WS. Children with WS had better language abilities than nonverbal abilities. Compared to TD children, those with WS exhibited delayed acquisition of first words, word combinations, and grammatical morphology. People with WS differed from those with other developmental language disorders in that vocabulary, and overall language skills were higher than expected relative to general cognitive capacities. Children with DS showed significant speech deficits and cognitive and language delays, but vocabulary skills were less impaired than grammatical abilities. Children with DS demonstrated slower overall language development than nonlinguistic development (nonverbal cognitive skills).
Vicari et al. (2005)		This study reported poorer verbal short-term memory skills in DS than in WS, while visual–spatial task performance was better in DS than in WS. Verbal short-term memory deficits in DS involved poor phonological and lexical-semantic processing of verbal information, while deficits in WS involved poor lexical-semantic information to verbal span. Individuals with WS were more impaired in the temporary retention of visual–spatial information than visual-object information, whereas individuals with DS did poorly in tasks requiring both types of material. In WS, the deficiency appeared to be mnesic, while in DS, the deficit appeared connected to difficulties with perceptual processing rather than dysfunctional memory. The study concluded that individuals with WS exhibit distinct difficulties in visual–spatial memory but not visual-object working memory compared to persons with DS, while individuals with DS do poorly in both visual–spatial and visual-object tasks.
Vicari (2006)		Individuals with WS demonstrated impaired learning of visual–spatial material but typical learning of visual-object patterns compared to TD children of the same mental age, while individuals with DS demonstrated typical visual–spatial sequence learning but impaired learning of visual-object patterns.
Jarrold et al. (2007)		This study reported relatively selective verbal short-term memory problems in DS but no differentiated abnormalities in long-term memory for verbal information. Long-term memory for visual information was poor in both DS and WS. The study concluded that the visual–spatial short-term memory deficiencies in WS are secondary to more general visuospatial processing problems.
Price et al. (2007)		This study evaluated the receptive vocabulary, grammatical morphology, and syntactic abilities of boys with FXS (who were also categorized as having autism, autistic spectrum disorder, or no autism), boys with DS, and TD boys at comparable nonverbal developmental stages. After adjusting for nonverbal cognition and maternal education, the three FXS groups did not differ in language comprehension but scored lower than TD boys. Boys with DS showed poorer performance in language comprehension tasks than non-autistic boys with FXS and TD boys. Scores for receptive vocabulary, grammatical morphology, and syntax did not differ among FXS, DS, and TD groups. Boys with FXS and DS demonstrated distinct language deficiency profiles.
Cornish et al. (2007)		This study also reported a complex cognitive profile in children with FXS. Children with FXS typically presented with social anxiety, hyperarousal, autistic-like features, and delayed language development. People with FXS demonstrated greater impairments in visual working memory than verbal working memory, while the reciprocal pattern was found in DS. Persons with FXS had deficits in visuomotor skills, difficulties acquiring math skills, and attention deficits.
Vicari et al. (2005)		This study compared implicit memory processes in people with WS or DS to test ‘etiological specificity’ assumptions about the skill acquisition capacities of people with developmental delays. People with WS and DS presented with developmental delays but different patterns of procedural learning. In the Serial Reaction Test, people with WS demonstrated inadequate implicit learning of the temporal sequence of events. Skill learning was better in DS than in WS.
Ypsilanti and Grouios (2008)		This review concluded that people with WS have superior linguistic skills (e.g., expressive vocabulary) compared to individuals with DS, but there is a significant difference in receptive vocabulary. Visual–spatial abilities are poorer, but verbal short-term memory skills are better in children with WS than with DS. During the first two years of life, language development does not differ between DS and WS.
Martens et al. (2008)		This review described the WS cognitive profile as a severe disconnect between preserved expressive language, face processing skills, and markedly impaired visuospatial skills. Most people with WS have Full-Scale IQ scores in the 50–60 range. Language development appears to be typical (although delayed) in syntax, semantics, word fluency, and expressive vocabulary, but grammatical comprehension, gender agreement, pragmatics, and oral fluency are poor. People with WS also display hypersocialability, hyperactivity, and anxiety.
Chapman (2008)		This was a review of the book ‘Speech and Language Development and Intervention in Down Syndrome and Fragile X Syndrome’. The authors state that children with DS have significant impairments in expressive vocabulary and syntax but relative strengths in pragmatic abilities, gestural communication, and understanding. In contrast, boys with FXS are typically impaired in pragmatics, receptive language, and expressive language, although they exhibit relative strengths in vocabulary.
Kogan et al. (2009)		People with DS exhibited weaknesses in egocentric spatial learning and object discrimination tasks and relative impairments in visual-perceptual and visual–spatial reversal learning. People with FXS performed relatively poorly on tests of object discrimination learning and reversal learning but demonstrated superior object recognition memory, egocentric spatial learning, and reversal learning compared to the DS group. DS and FXS groups performed relatively poorly on visual–spatial working memory tasks.
Conners et al. (2011)		This review identified the many memory impairments connected to intellectual disability and the effects of these impairments on reading development in DS, WS, and FXS. Word recognition skills in DS are likely assisted by relatively good immediate visual memory and rapid phonological retrieval, while poor verbal working memory skills are likely to due to difficulties with phonological recoding and reading comprehension In WS, relatively preserved visual and verbal working memory and quick phonological retrieval may allow for reasonably good word and nonword reading abilities. Severe impairments in visual and verbal working memory, learning, and storytelling predict difficulties with word recognition and reading comprehension in FXS.
Carney et al. (2013a)		People with WS showed relatively poor visuospatial short-term memory skills compared to individuals with DS.
Carney et al. (2013b)		Individuals with WS demonstrated relative visuospatial deficits as evidenced by poorer performance in executive-loaded working memory (ELWM) and fluency tests compared to TD individuals. Individuals with DS exhibited relative verbal difficulty in the set-shifting domain. Furthermore, DS and WS populations showed pervasive deficits across modalities in one specific domain, ELWM for DS and inhibition for WS. Both WS and DS groups showed executive function (EF) difficulties compared to the TD group. However, executive performance was affected differently by the EF task type (verbal/visuospatial) and EF domain.
Costanzo et al. (2013)		This study demonstrated several executive function impairments in DS and WS, including poor working memory, visual categorization, auditory selective attention, visual inhibition, and sustained visual attention. However, abilities in other executive functions differed between DS and WS groups. Participants with DS performed poorly on shifting, verbal, memory, and inhibition tasks, whereas participants with WS performed poorly on planning tasks.

WS, William’s syndrome; DS, Down syndrome; FXS, Fragile X syndrome; DS22q11.2, 22q11.2 deletion syndrome; IQ, Intelligence Quotient; SD, Standard deviation; TD, Typically developing; EF, Executive function.

Alternatively, Ypsilanti and Grouios (2008) reported that the cognitive profile of DS is more similar to that of WS, although others have not reported such similarities (Carney et al., 2013a). These similarities include strengths in similar language domains and shared deficiences in visuospatial ability (Bellugi et al., 2000; Atkinson et al., 2001; Vicari et al., 2005; Carney et al., 2013b). However, characterizing the cognitive profile of WS and DS in terms of a dissociation between language and visuospatial abilities is an oversimplification as both syndromes exhibit unequal strengths and weaknesses within each cognitive domain (Vicari, 2001; Rice et al., 2005; Menghini et al., 2011; Costanzo et al., 2013). The typical cognitive profile of WS includes relatively intact language and facial processing skills but severely impaired visual–spatial skills (Jernigan et al., 1993), in contrast to DS. Language development in WS appears to be typical in syntax, semantics, word flow, and expressive vocabulary (albeit delayed; Ypsilanti et al., 2005; Ypsilanti and Grouios, 2008). Conversely, deficits were observed in grammatical comprehension, gender matching, pragmatics, and oral fluency (Martens et al., 2008). Several studies have confirmed visual–spatial impairments in WS (Atkinson et al., 2001; Vicari et al., 2005; Jarrold et al., 2007). Individuals with WS also show significant behavioral problems, including hyperactivity, anxiety, and hypersociality (Martens et al., 2008). Immediate spatial recall, verbal and spatial delayed memory and learning, and semantic retrieval requiring proper names are also deficient in WS. However, relative strengths are instantaneous verbal and visual recall, visual-delayed memory and learning, and phonological retrieval. Weaknesses in visual–auditory learning and semantic retrieval, as well as relative strengths in instantaneous recall (both visual and verbal) and rapid phonological retrieval, are particularly significant influences on reading development (Conners et al., 2011).

Individuals with DS22q11.2 also exhibit a complex neurocognitive profile characterized by a variable IQ score, relatively poor language, computational power, visual–spatial processing skills, and visual-object short-term memory, as well as weaknesses in executive function, motor skills, psychosocial function, and working memory (Woodin et al., 2001; Sobin et al., 2005; Persson et al., 2006; Jacobson et al., 2010; Niklasson and Gillberg, 2010; Vicari et al., 2012; Morrison et al., 2020). Sobin et al. (2005) also reported severe visual attention and working memory impairments in DS22q11.2.

## 4. Discussion

We searched the literature over the past several decades to identify studies investigating regional brain volume abnormalities and cognitive deficits in children and adults with DS and comparing pathological features among DS, FXS, WS, and DS22q11.2. A synthesis of results from the 84 included studies indicates that DS is typically associated with reduced total brain volume and regional volume reductions within the hippocampal formation, frontal, parietal, temporal, and occipital lobes, brain stem, and cerebellum. However, morphometric, and cognitive abnormalities differed across studies, suggesting the influence of factors such as age, sex, and comorbidity profile. While there is no ‘stereotypical’ DS profile, this review identified several brain regions and cognitive domains with relatively stable differences compared to matched TD groups and compared to FXS, WS, and DS22q11.2 groups that may provide clues to disease pathogenesis and guidance for the development of more targeted and individualized therapeutic interventions.

Although DS is a neurodevelopmental condition exhibiting substantial changes in symptom profile with age, most of the reviewed neuroimaging studies, particularly those employing VBM, included adolescents and adults. Moreover, from middle life onwards, individuals with DS are at heightened risk of AD dementia, and even those without dementia exhibit significant age-related changes in regional brain volume (Kesslak et al., 1994; Krasuski et al., 2002; Teipel et al., 2004). Furthermore, according to Teipel and Hampel (2006), the specific effects of age may be obscured by more significant variability in brain morphology. Therefore, larger-scale studies of multiple well-delineated clinical subgroups are required to address the independent impact of aging on the DS brain, cognitive capacity, and behavior.

Despite heterogeneity within and across DS study groups, there were relatively consistent associations between working memory deficits and volume reductions within the parietal lobe (Menghini et al., 2011) and temporal lobe (Galaburda and Schmitt, 2003; Pennington et al., 2003; Vicari, 2006; Menghini et al., 2011). Further, reduced temporal lobe volume was strongly associated with language deficits, although some studies, such as Pinter et al. (2001b), reported a larger corrected temporal lobe volume in DS. Nonetheless, the balance of neuroimaging evidence suggests that a disproportionately smaller temporal lobe contributes to language deficits in DS. Also, Fabbro et al. (2002) proposed that language deficits are partly due to impairments in frontocerebellar structures involved in articulation and verbal working memory. However, we did not include frontocerebellar structures in our review.

There were also relatively stable associations between regional reductions in hippocampal volume and deficits in language and memory (Raz et al., 1995; Krasuski et al., 2002; Pennington et al., 2003). Moreover, the hippocampus is among the most vulnerable regions to damage by AD, including from the premature AD observed in many individuals with DS concomitant with dementia onset (Aylward et al., 1999; Hamadelseed et al., 2022). Less consistent was the association between age and hippocampal volume. Raz et al. (1995) and Aylward et al. (1999) found no correlation between age and hippocampal volume, while Kesslak et al. (1994) found a significant correlation. A decrease in hippocampal volume with age may help explain the learning and memory deficits that start in infancy, continue throughout childhood, and frequently worsen in adulthood (Kates et al., 1997b). We speculate that deficits in hippocampal circuit function and plasticity undetectable by structural neuroimaging contribute to specific language and memory deficits in DS. Similarly, long-term memory impairments may be linked to medial temporal lobe volume reductions or circuit dysfunction. Higher-resolution functional neuroimaging studies are needed to elucidate these mechanisms.

The memory profile observed in DS appears distinct from that of the other cognitive impairment-related hereditary diseases reviewed. For instance, verbal and visual short-term memory is relatively preserved. In contrast, spatial working memory is relatively deficient in WS, and a similar pattern is observed in explicit long-term memory among adolescents with WS. Conversely, individuals with WS demonstrate reduced implicit process learning capacity (Vicari, 2006), while this form of learning is relatively intact among individuals with DS.

A likely reason for the variability observed within and across studies is the small individual sample size and limited statistical power (Deinde et al., 2021). However, recruiting large numbers of individuals with neurodevelopmental disorders, especially children, for comprehensive neuropsychological testing and neuroimaging is challenging. First, many people (regardless of cognitive status) are afraid of brain scans (Enders et al., 2011), while MRI acquisition may be challenging for people with DS as many are obese and thus may experience claustrophobia (Melville et al., 2005; Head et al., 2018). Furthermore, individuals with DS may experience discomfort in the prone position during extended periods due to the disorder’s distinctive neck and facial anatomy (Head et al., 2018). Third, fear of scanning may be further compounded by an inability to understand the purpose of the procedure or follow instructions. Such individuals may find it difficult to remain stationary during the scanning procedure, resulting in movement artifacts (Neale et al., 2018). While sedation may be helpful, it may also influence the neural processes under study (Landt et al., 2011; Head et al., 2018). Allowing the DS participants to visit the scanning site and become familiar with the scanning technique may help alleviate fear and promote relaxation and trust in the operator. Mock scanners and shorter scan wait times may also help set participants at ease.

A general problem limiting the sample size for neuroimaging is the operating expense and equipment availability (Neale et al., 2018). For this reason, MRI should be combined with cognitive and clinical tests to maximize data acquisition and provide an independent data stream for better interpretation. It is also necessary to compare neuroimaging results between people with DS with and without dementia. Such information could help identify regions most vulnerable to AD, predictive biomarkers, and underlying pathogenic mechanisms for effective treatment.

## 5. Conclusion

This review is one of the few to assess the unique neuroanatomic and neuropsychological characteristics of Down syndrome comprehensively and critically. A synthesis of the included studies suggests that cognitive deficits in DS, especially within language and memory domains, may be associated with abnormalities in regional brain volume. Indeed, DS is characterized by relatively consistent GM and WM loss in regions subserving memory and language, including the hippocampal formation and lateral temporal lobe. Thus, deficits in memory and language are more severe than expected from global intellectual dysfunction. In addition, a reduction in parietal lobe volume may be a significant predictor of cognitive deficits in DS, particularly among individuals with impaired visuospatial processing ability. Neuroimaging is a powerful technique that can help researchers detect early structural, functional, neurochemical, and metabolic alterations associated with DS, including AD development. These neuroimaging discoveries could allow physicians to treat high-risk patients before AD onset, thereby maintaining cognitive function and quality of life. Neuroimaging in DS may also facilitate the development of new therapies and improved treatment response monitoring. Large-scale longitudinal studies will be critical for expanding our understanding of AD pathogenesis in DS.

In summary, this review provides educational psychologists and instructors with crucial information to identify and address the various learning and social challenges experienced by individuals with DS. Understanding the cognitive and behavioral phenotypes of DS will help professionals and parents better understand and manage the condition in daily life, helping people with DS achieve their highest level of independent function.

## Data availability statement

The original contributions presented in the study are included in the article/supplementary material, further inquiries can be directed to the corresponding author.

## Author contributions

OH, MC, MW, and TS performed material preparation, data collection, and analysis. OH wrote the first draft of the manuscript, and all authors commented on previous versions. All authors contributed to manuscript revision, read, and approved the submitted version.

## Funding

This research project was made possible through funding from the Friedrich-Naumann Foundation for Freedom (Scholar-Number: 8683/P611, 2021).

## Conflict of interest

The authors declare that the research was conducted in the absence of any commercial or financial relationships that could be construed as a potential conflict of interest.

## Publisher’s note

All claims expressed in this article are solely those of the authors and do not necessarily represent those of their affiliated organizations, or those of the publisher, the editors and the reviewers. Any product that may be evaluated in this article, or claim that may be made by its manufacturer, is not guaranteed or endorsed by the publisher.

## Footnotes

^1^ https://rarediseases.info.nih.gov/diseases/10247/down-syndrome

^2^ http://www.intellectualdisability.info/diagnosis/articles/the-genetics-of-downs-syndrome

^3^ https://www.ndss.org
